# The microbiological effect of virgin coconut oil on the morphological and volumetric dimensional changes of 3D printed surgical guides (in vitro study)

**DOI:** 10.1186/s12903-022-02671-8

**Published:** 2022-12-23

**Authors:** Rania T. Khalil, Ahmed Alshimy, Eglal Elsherbini, Mervat E. Abd-ELLAH

**Affiliations:** 1 Department of Prosthodontics, Faculty of Dentistry, Alexandria University, Alexandria, Egypt; 2 Department of Microbiology, Medical Research Institute, Alexandria University, Alexandria, Egypt

**Keywords:** Microbiological effect, Virgin coconut oil, Dimensional changes, 3D printing, Surgical guides

## Abstract

**Background/objectives:**

Disinfection of surgical guides is mandatory for intraoperative use. Virgin Coconut Oil may be a potent alternative disinfectant; however, its effect has not been fully discussed in dentistry. The objectives of this study were to compare the morphological and the volumetric dimensional changes of 3D printed surgical guides after immersion in three disinfectants: 100%Virgin Coconut Oil, 2% Glutaraldehyde, and 70% Ethyl Alcohol and to assess the antimicrobial effectiveness of the tested disinfectants.

**Materials and methods:**

A surgical guide was designed using open platform software to print thirty guides and then cut them into two halves (N = 60). Pre-disinfection scans of the first half of the three study groups (n = 30) were performed using Cone-beam Computed Tomography, then immersed for 20 min in three disinfectants as follows: group VCO was immersed in 100% Virgin Coconut Oil, group GA was immersed in 2% Glutaraldehyde, and group EA was immersed in 70% Ethyl Alcohol. Post-disinfection scans of the first half of the three study groups (n = 30) were performed and then compared morphologically and volumetrically using an analyzing software program The second half of the three control groups (n* = 30) were soaked for 20 min in sterile distilled water as follows: group VCO*, group GA*, and group EA* for the assessment of the antimicrobial effectiveness of the three tested disinfectants.

**Results:**

At the morphological assessment of the dimensional changes, group VCO were the most accurate with the lowest mean deviation value of 0.12 ± 0.02 mm and root mean square value of 0.12 mm, group GA and group EA were less accurate with mean deviation value of = 0.22 ± 0.05 mm and = 0.19 ± 0.03 mm and root mean square value of 0.22 and 0.20 respectively (*p* < 0.001). At the volumetric assessment, group VCO showed lower volumetric changes with a mean deviation value of 0.17 ± 0.10 mm, root mean square value of 0.19 mm, than group GA with mean deviation value of 0.23 ± 0.10 mm, root mean square value of 0.25 mm and group EA with mean deviation value of 0.27 ± 0.11 mm, root mean square value of 0.29 mm, however, no statistically significant differences were found between the three study groups (*p* = 0.10). The antimicrobial effectiveness of the three tested disinfectants showed a hundred percent (100%) reduction in the total microbial count in the first half of the three study groups treated with the three disinfectants revealing no bacterial growth, however, statistically significant differences were found between the second half of the three control and the first half of the three study groups. (*p* < 0.001).

**Conclusions:**

Virgin Coconut Oil showed higher morphological dimensional accuracy of the tested surgical guides than Glutaraldehyde and Ethyl Alcohol without causing any volumetric dimensional changes in the 3D printed surgical guides after disinfection for 20 min and the antimicrobial effectiveness was the same between the three tested disinfectants without showing any microbial growth.

**Supplementary Information:**

The online version contains supplementary material available at 10.1186/s12903-022-02671-8.

## Introduction

The long-term success of dental implants is directly related to proper diagnosis and good treatment planning for the ideal implant position and precise transfer of the planned position to the surgical site [1]. The conventional method of placing an implant was proven to be a complicated, inaccurate laboratory procedure and difficult in placing the implant fixture, as planned [1, 2]. Currently, additive manufacturing or 3D printing is commonly used in dentistry, with its most extensive application being the fabrication of computer‐aided design/computer‐aided manufacturing (CAD/CAM) surgical guides which played a crucial role in dental implant surgery to allow the performance of surgical and prosthetic treatments with great accuracy of implantation and final restoration [3]. Accordingly, the strategic position of the implant is transferred to the surgical area utilizing a surgical guide which enables predictable and minimally invasive surgery [4]. The Glossary of Prosthodontic Terms defines a surgical template (or surgical guide) as “a guide used to assist in proper surgical placement and angulation of dental implants [5].

The fabrication of surgical guides usually follows a fully digital computerized workflow that does not include the standard multiple traditional phases [6]. Digital implant guide production includes the following steps: data acquisition from patients’ oral cavity and bone, digital model preparation using virtual planning software, and stereolithographic guide production via a prototyping system, thus, allowing virtual implants to be placed in an ideal, prosthetically driven manner [7].

The surgical guide represents the union of guiding cylinders (sleeves) and contact surface [8]. Angulation and depth of implant osteotomy are controlled by guided surgical drills through a metal sleeve embedded in the surgical guide. Hence, this cylinder helps in transferring the plan by guiding the drill in the exact location and orientation [9].

Moreover, the surface of the guide usually comes in direct contact with bone, and blood, and poses a potential risk of pathogenic transmission [10]. Bacterial infection is not a common reason for early implant failure or surgical complications if stringent aseptic surgical protocols are followed. However, knowing that cross-contamination inevitably occurs in many dental laboratories, the clinician must be aware of and follow proper protocols for maintaining a sterile surgical field to ensure minimal post-operative complications [11].

Correspondingly, like all other instruments used in implant surgery, disinfection of surgical guide is of the utmost importance for the most optimal outcome where the success of the implant placement will be monitored, as well as the safety and health of the dentist, dental technician, and patient [10]. Notably, one of the considerable obstacles to the utility and efficacy of 3D printed surgical guides is the disinfection of these thermosensitive devices. Owing to the heat intolerance and the porous nature of resin material from which stereolithographic surgical guides are made, they may undergo deformation during steam sterilization [10–12].

According to guidelines of the Center for Disease Control and Prevention (CDC), medical instruments are divided into three distinctive categories; critical, semi-critical, and non-critical—pertaining to the extent to where instruments may have the probability of infection transmission [13]. With that being said, the surgical guide is classified as semi-critical in which decontamination occurs most often utilizing high-level disinfectants, such as 2% Glutaraldehyde (GA) and 70% Ethyl alcohol (EA) for 15–20 min before surgery [13]. There are always drawbacks with such chemical disinfectants that have a potential cause for concern to be in such proximity to the skin and have hazardous effects on the environment when released through evaporation [14]. Even though, EA has demonstrated more favorable effects in antimicrobial achievement in comparison with Chlorhexidine, it is immensely flammable, must be stored in a cool place, irritates the oral tissues, and is not effective against non-enveloped viruses or spores. While GA is toxic, a skin irritant, and harsh to mucous membranes, must be used in well-ventilated areas and is not recommended as a spray or solution for the decontamination of surfaces [15–17]. Consequently, scientists actively seek out alternative products for the implementation of effective disinfection protocol. Moreover, the increasing resistance of human pathogenic microorganisms to the traditional means of treatment urged natural herbal products to receive substantial consideration over the past three decades [18]. Most recently, VCO has gained popularity as a distinct dietary oil because it consists mainly of medium-chain fatty acids affecting its physical and chemical properties. Additionally, it contains 92% saturated fatty acids, 50% of which is lauric acid with antimicrobial, antibacterial, antifungal, antiviral, anti-inflammatory and antinociceptive, and antioxidant effects against a wide variety of microorganisms [19–22]. As it was proven, VCO has exceptional antimicrobial activity when compared to other edible oils due to its high saponification index (254.82 mg KOH/100 g) leading to a reduction in microorganism accumulation and employing a powerful cleansing effect [23].

Eventually, the antibacterial effect of the VCO is attributed not only to the lower acidic pH nature—between 2.52 and 4.38 which generally increases lauric acid activity—but, also to the medium-chain mono-glycerides as monolauric and monocarpic acid which destroy a wide variety of lipid-coated bacteria by disrupting their lipid membranes and inhibiting the enzymes involved in energy production and nutrient transfer [24, 25]. Besides that, the high viscosity of the VCO reduces the aggregation and adhesion of microorganisms which has a great ability to inhibit the growth of Candida Albicans [23]. VCO is remarkably very effective against many viruses due to its effective disintegration of the virus particles which disrupts its maturation; hence, preventing the binding of viral M proteins to the host cell membrane [26]. Also, the anti-inflammatory activity of VCO is outstanding by inhibiting the synthesis of inflammatory mediators responsible for the formation of pain and edema. Furthermore, the anti-nociceptive property of VCO inhibits the proliferative phase during the inflammatory process [24, 27]. VCO is extracted directly from fresh, mature coconut kernel without going through a refined process. This preserves the natural organic active compounds with antioxidant properties that accelerate the healing process of damaged tissues by promoting re-epithelialization and collagen synthesis [23, 25–28].

The clinical necessity for chemical-free, effective, convenient, and tissue-friendly disinfectant, which inhibits the growth of microorganisms without affecting the dimensional accuracy or the surface details of the surgical guides, has been long overlooked. Hence, VCO was selected as an alternative natural disinfectant for the decontamination of the heat-sensitive surgical guides, however, its prospective as a disinfectant has yet to be thoroughly investigated. It is worth noting that many articles were related to the application of 3D printed devices in dentistry; still, the technique and effect of disinfection on the surgical guides, in conjunction with the evaluation of the possible dimensional changes, were scarcely addressed.

### Objectives

This study was carried out to compare the morphological and the volumetric dimensional changes of 3D printed surgical guides when immersed in three different disinfectants namely; 100% of VCO, 2% GA, and 70% EA solutions and to assess the antimicrobial effectiveness of these disinfectants used to decontaminate the respective surgical guides. The null hypothesis would show no significant differences in the morphological, or the volumetric dimensional changes of 3D printed surgical guides nor in the antimicrobial effectiveness of the three disinfectants after immersion in 100% VCO, 2% GA, and 70% EA solutions.

## Material and methods

### Study setting

This in vitro comparative study was held at the Department of Prosthodontics, Faculty of Dentistry, and the Department of Microbiology, Medical Research Institute, Alexandria University. Prior to commencement, all the methods were approved by the Research Ethics Committee at the Faculty of Dentistry, Alexandria University, Egypt (IRB 00010556–IORG 0008839) after ensuring that all methods are in accordance with the Helsinki declaration.

### Sample size calculation

The minimal sample size was calculated based on a previous study aimed to investigate the effect of steam heat sterilization on the dimensional accuracy of surgical guides. Marei et al., [29] concluded that steam heat sterilization has a non-significant effect on the dimensional changes of the tested surgical guides. Based on their results, adopting a power of 80% to detect a standardized effect size in the dimensional accuracy (d = 0.9102) (large-sized standardized effect size), and level of significance 95% (alpha = 0.05), the minimum required sample size was found to be 60 surgical guides (30 surgical guides cut into two halves, number of groups per each half = 3, and number of surgical guides per group = 10 [30]. Any error in the procedure that may lead to the loss of any sample (guide) was compensated by replacing the lost guide with a new one to maintain the required minimum sample size of 60 and to control for attrition (withdrawal) bias [31].

### Study design

**Fig. 1:**
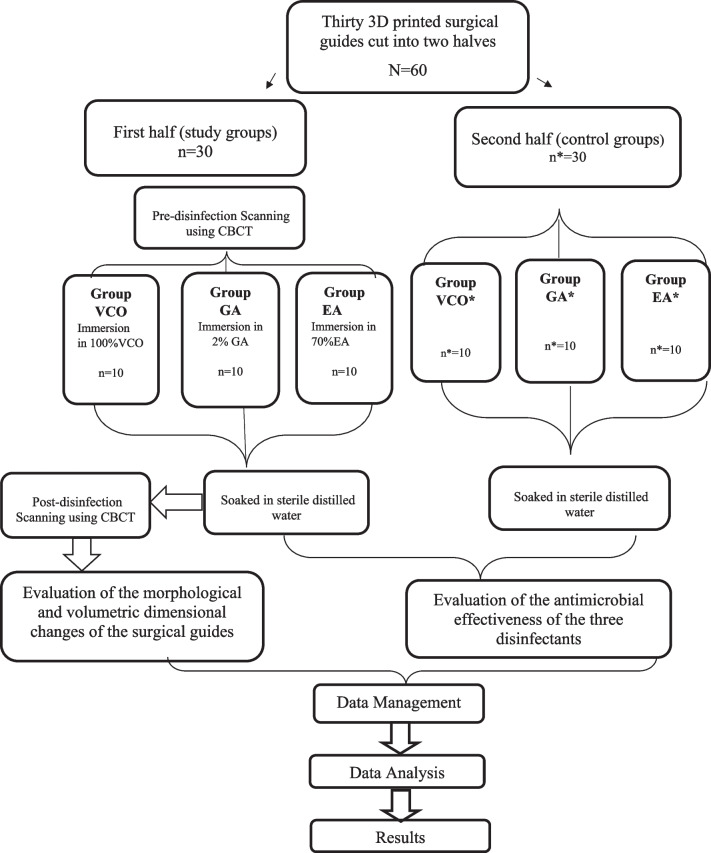
Flow chart of the study design

Thirty identical surgical guides were printed and cut into two halves (N = 60). The first half (n = 30) was sub-grouped into three study groups as follows: group VCO (n = 10), group GA (n = 10), and group EA (n = 10) which was scanned before the disinfection process using a low dose, high-resolution Cone-beam Computed Tomography (CBCT), then immersed for 20 min in one of three disinfectants which were, 100%VCO, 2% GA, and 70% EA. After the disinfection process, each first half of the three study groups was soaked in sterile distilled water then scanned using the same settings and same parameters and assessed morphologically and volumetrically. The second half (n* = 30) was sub-grouped into three control groups as follows: group VCO*(n* = 10), group GA*(n* = 10), and group EA*(n* = 10) which was soaked in sterile distilled water for 20 min for the assessment of the antimicrobial effectiveness of the three tested disinfectants (Fig. 1).

### Surgical guides production

**Fig. 2:**
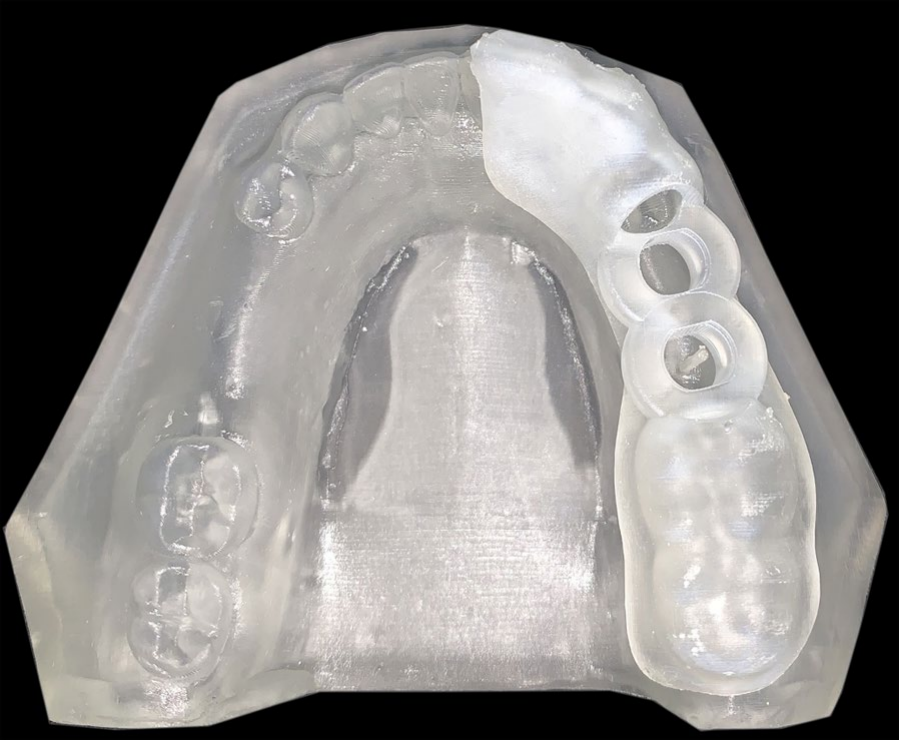
First half of the surgical guide placed accurately on 3D printed study model with 1 mm diameter fixed marker

A surgical guide was fabricated by making an impression of a mandibular dental arch with bilateral missing second premolars and first molars using polyether impression material and poured with dental stone to obtain a study cast [32, 33]. The study cast was scanned using CBCT (Acteon, X-mind Trium, Italy). The DICOM (Digital Imaging and Communications in Medicine) data were exported as a Standard Tessellation Language (STL) file to create a 3D model where the surgical guide was virtually designed using an open platform software (Blue Sky). Ten identical surgical guides were printed three times in a clear photoreactive resin material (Resin cartridge form2; Formlabs Inc) using a desktop stereolithography 3D printer (formlabs 2, Formlabs Inc, Somerville, MA, USA) with the following parameters; layer thickness = 0.1 mm, layers number = 672, layer volume = 103.74 ml, offset (block out undercuts offset) = 0.15 and printing time = 6 h 45 min [34]. The 3D printing works by adding layers of curable liquid photopolymer onto a build tray where fine layers accumulated to create 3D surgical guides which were rinsed in a bath of 90% Isopropyl Alcohol for 10 min and then inserted in a bath of clean, unused, 90% Isopropyl Alcohol. The printed guides were left to dry for an additional 10 min and then exposed to 72 watts of Blue Ultraviolet light oven (315–400 nm) for 10 min at 60 °C according to the manufacturer’s instruction, to achieve optimal mechanical properties. The support material was removed after curing using the flush cutter included in the Formlabs Standard Finish Kit. One study model with a 1 mm diameter fixed marker was also printed using the same material and the same 3D printer. The surgical guides were then cut into two halves using a sterile cutting disk at low speed and placed onto 3D printed model to be checked for accurate fitting by an operator [35, 36]^.^ (Fig. 2; Additional file 1).

Pre-disinfection CBCT scans of the three study groups were performed using low dose and high-resolution protocol at a proper field of view with the following settings; tube 5 current: 6 mA, potential: 80 kV, voxel size: 0.1 mm, and the images were exported as STL files. After that, each half of the three study groups was immersed into one of three disinfectants for 20 min and then soaked in sterile glass containers containing 100 ml. of sterile distilled water. Post-disinfection CBCT scans were performed using the same settings and device for second images to be exported as second STL files. The DICOM files of both scans were exported from the CBCT as two STL files along with one STL file for the digital model and imported into interactive CAD software for the analysis of the morphological and the volumetric dimensional changes. In each study group, the pre-disinfection guides were registered as the control (actual data) and the post-disinfection guides were set as study groups (nominal CAD data).

### Analysis of the morphological changes

**Fig. 3:**
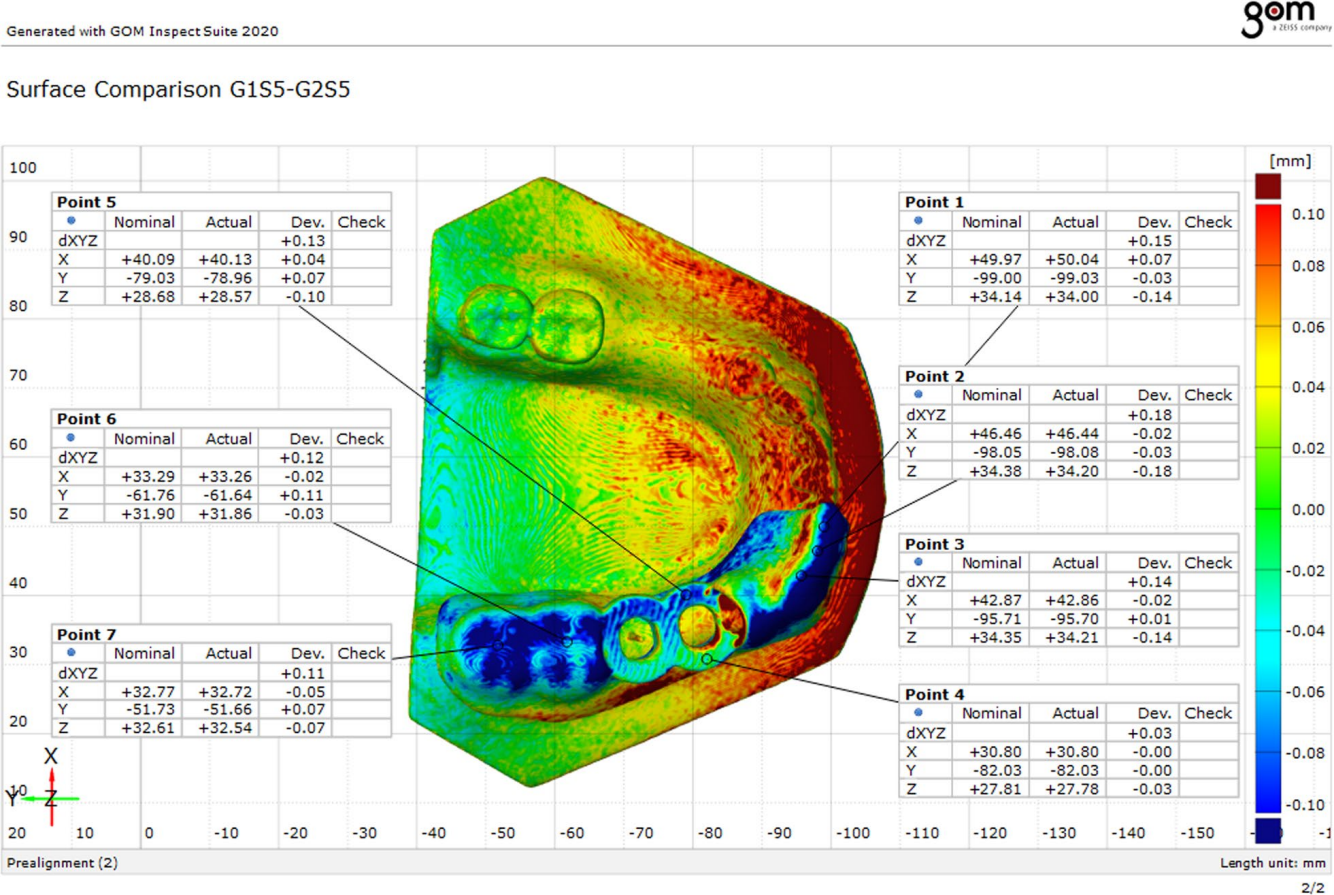
Morphological assessment of dimensional changes at seven points selected on the surface of the first half of the surgical guide where the pre-disinfection guides were set as the control (actual data) and the post-disinfection guides were set as the study group (nominal CAD data)

3D analyzing CAD software (GOM Inspect) was used for the morphological assessment through the surface comparison analysis where a curve was drawn on the surface of the surgical guides before disinfection and seven points were indicated. The same points were also indicated on the corresponding positions after disinfection. Then each post-disinfection guide was superimposed on the pre-disinfection one with the help of the 1 mm diameter reference marker which was fixed and attached to the 3D printed model. At each point, the deviation in x, y, and z axes(dxyz) was measured to calculate the mean, the standard deviation (SD), and the root mean square (RMS) between the three study groups before and after the disinfection process [29, 37] (Fig. 3).

### Analysis of the volumetric changes

**Fig. 4:**
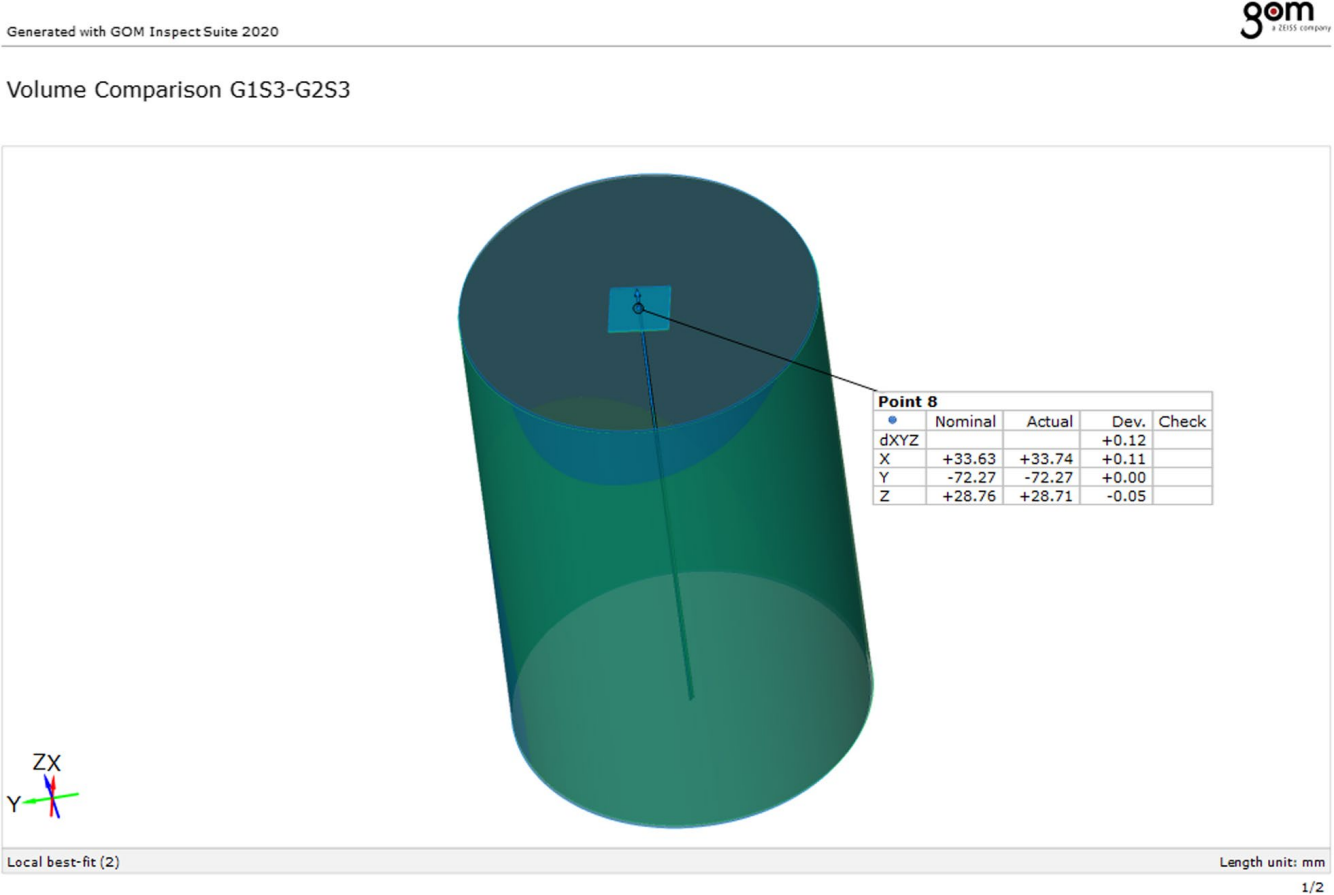
Virtual cylinder produced after superimposition and alignment of the two pre-and post-disinfection STL files of surgical guides for the volumetric assessment where the software determined point and plane at the center of the cylinder, which represented the sleeve space. Blue and green geometries referred to pre- and post- disinfection surgical guides, respectively

A virtual cylinder produced by the 3D analyzing CAD software (GOM Inspect) was fitted into the sleeve space. The software determined the center of the cylinder represented by a point{8} related to the reference marker which was fixed to the 3D printed model so, accurate superimposition and alignment of the 2 STL files were achieved. This point which intersected the center of the cylinder and the plane at the top of the sleeve space projected from the pre-disinfection guide, was superimposed to the post-disinfection guide. The software measured the deviation in x, y, and z to calculate the mean, the standard deviation (SD), and the root mean square (RMS) between the three study groups before and after the disinfection process [29–37] (Fig. 4).

### Microbiological trial

All surgical guides were investigated at different time intervals; after one day, one week, and two weeks from the production stage. Each first half of the three study groups was immersed in one of the three disinfectants; 100%VCO, 2% GA, and 70% EA for 20 min, left to dry for an additional 10 min then soaked in sterile glass containers containing 100 ml. of sterile distilled water for 10 min, and each second half of the three control groups was immersed in sterile glass containers containing 100 ml. of sterile distilled water for 20 min. Three samples were pipetted and cultured on three microbiological media, in which 50 µl (µl) were spread over the surface of Blood (Oxoid, CM0271) and MacConkey agar plates (Oxoid, CM0115) to be incubated at 37 °C for 24 h, and over the surface of Sabouraud dextrose agar plates (HiMEDIA, M063) to be incubated at 37 °C for 48–72 h. After the incubation period, all plates were examined, and the microbial count was done and expressed as colony-forming units per plate (CFU/plate). The percentage (%) of reduction was calculated by the following equation [18]:

$$\% \;{\text{of}}\;{\text{reduction}} = {\text{No}}\;{\text{of}}\;{\text{CFU}}/{\text{control}}\,{\text{plate}} - {\text{CFU}}/{\text{study}}\;{\text{plate}}/{\text{No}}\;{\text{of}}\;{\text{CFU}}/{\text{control}}\;{\text{plate}} \times 100$$

### Statistical analysis

The comparison of the morphological and the volumetric dimensional changes between the groups was analyzed using the One-Way ANOVA test that was used to verify the normality for all variables using descriptive statistics, plots, and normality tests. All variables showed normal distribution, so means, SD, and RMS were calculated. The level of statistical significance was set at *p* < 0.05. Data were analyzed with IBM SPSS statistical software V 23.0, (SPSS Inc).

## Results

### Analysis of morphological changes

At the morphological assessment, statistically significant changes were found between the three study groups; group VCO with mean deviation = 0.12 ± 0.02 mm and RMS = 0.12, group GA with mean deviation = 0.22 ± 0.05 mm and RMS = 0.22 mm, and group EA with mean deviation = 0.19 ± 0.03 mm and RMS = 0.20 (*p* < 0.001) (Table 1). Multiple pairwise comparisons were carried out when the One-Way ANOVA test was significant using Bonferroni-adjusted significance levels. Group GA versus group EA comparison showed no statistically significant changes in accuracy with *p* = 0.51, while group VCO versus group GA and group EA comparisons showed statistically significant changes in accuracy with *p*** < **0.001, *p*** = **0.001 respectively (Table 2, Figs. 8, 10).

**Table 1:** Morphological assessment of dimensional changes at seven points between the three study groups pre- and post-disinfection

	Group VCO	Group GA	Group EA	*p*
*Point 1*				
Mean ± SD	0.11 ± 0.04	0.20 ± 0.07	0.25 ± 0.06	< 0.001**
RMS	0.12	0.21	0.26	
*Point 2*				
Mean ± SD	0.11 ± 0.06	0.21 ± 0.09	0.23 ± 0.10	0.008**
RMS	0.12	0.23	0.24	
*Point 3*				
Mean ± SD	0.14 ± 0.04	0.22 ± 0.05	0.20 ± 0.08	0.02**
RMS	0.14	0.23	0.22	
*Point 4*				
Mean ± SD	0.12 ± 0.06	0.26 ± 0.06	0.25 ± 0.06	< 0.001**
RMS	0.13	0.26	0.26	
*Point 5*				
Mean ± SD	0.14 ± 0.04	0.22 ± 0.05	0.18 ± 0.04	0.001**
RMS	0.14	0.22	0.18	
*Point 6*				
Mean ± SD	0.12 ± 0.02	0.21 ± 0.13	0.13 ± 0.05	0.04**
RMS	0.12	0.25	0.14	
*Point 7*				
Mean ± SD	0.12 ± 0.03	0.18 ± 0.17	0.10 ± 0.05	0.25
RMS	0.12	0.24	0.11	
*Average of 7 points*				
Mean ± SD	0.12 ± 0.02	0.22 ± 0.05	0.19 ± 0.03	< 0.001**
RMS	0.12	0.22	0.20	

*SD* Standard deviation, *RMS* Root mean square

**Statistically significant at *p* < 0.05

**Table 2:** Pairwise comparisons using Bonferroni adjusted significance level

Groups	Compared to	*p*
*Point 1*		
Group VCO	Group GA	0.006**
	Group EA	< 0.001**
Group GA	Group EA	0.18
*Point 2*		
Group VCO	Group GA	0.04**
	Group EA	0.01**
Group GA	Group EA	1.00
*Point 3*		
Group VCO	Group GA	0.02**
	Group EA	0.09
Group GA	Group EA	1.00
*Point 4*		
Group VCO	Group GA	< 0.001**
	Group EA	< 0.001**
Group GA	Group EA	1.00
*Point 5*		
Group VCO	Group GA	0.001**
	Group EA	0.15
Group GA	Group EA	0.15
*Point 6*		
Group VCO	Group GA	0.049**
	Group EA	1.00
Group GA	Group EA	0.12
*Point 7*		
Group VCO	Group GA	0.66
	Group EA	1.00
Group GA	Group EA	0.34
*Average of 7 points*		
Group VCO	Group GA	< 0.001**
	Group EA	0.001**
Group GA	Group EA	0.51

**Statistically significant at *p* < 0.05

### Analysis of volumetric changes

At the volumetric assessment, no statistically significant difference was found between the study groups at the center of the sleeve space represented by point {8}, as group VCO showed less volumetric changes with mean deviation = 0.17 ± 0.10 mm, RMS = 0.19 mm, than group GA with mean deviation = 0.23 ± 0.10 mm, RMS = 0.25 mm and group EA with mean deviation = 0.27 ± 0.11 mm, RMS = 0.29 mm (*p* = 0.10) (Table 3, Figs. 9, 10).

**Table 3:** Volumetric assessment of dimensional changes between the three study groups pre-and post-disinfection

	Group VCO	Group GA	Group EA	*p* value
*Point 8*				
Mean ± SD	0.17 ± 0.10	0.23 ± 0.10	0.27 ± 0.11	0.10
RMS	0.19	0.25	0.29	

Statistically significant at *p* value < 0.05

### Microbiological trial

**Fig. 5:**
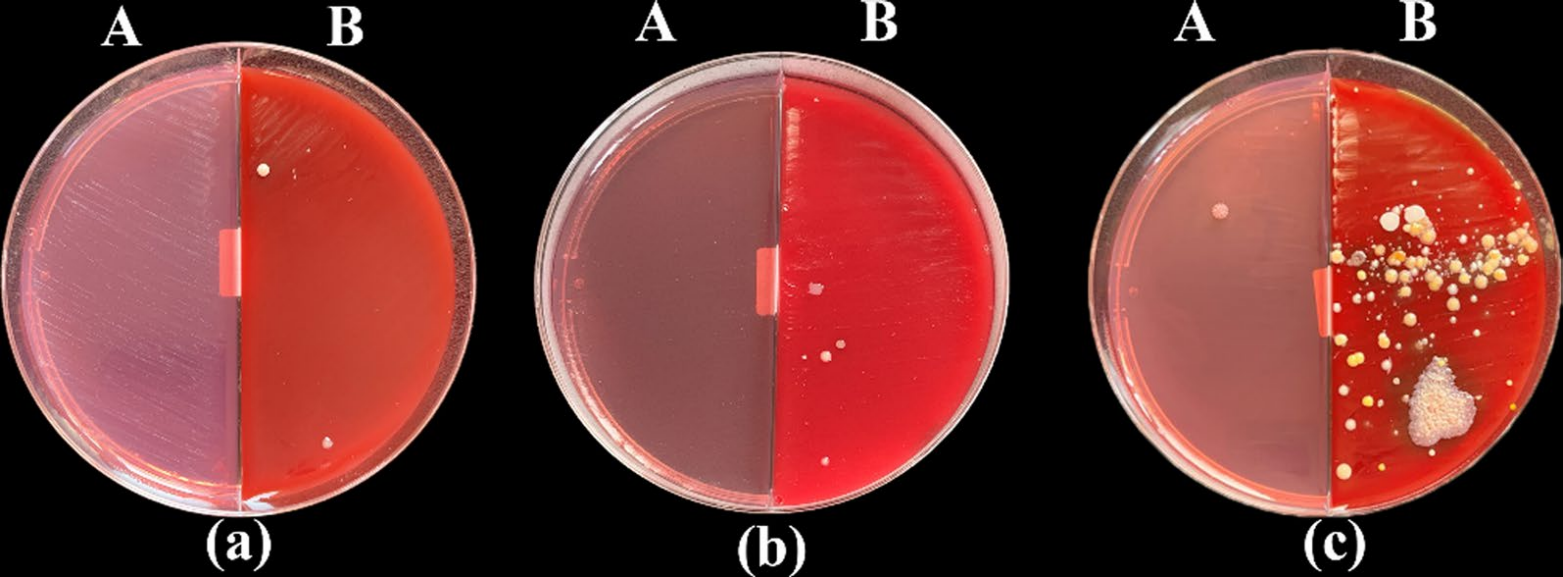
Culture results of the control group on MacConkey (A) and Blood (B) agar plates after one day (**a**), one week (**b**) and two weeks (**c**) from the production stage

**Fig. 6:**
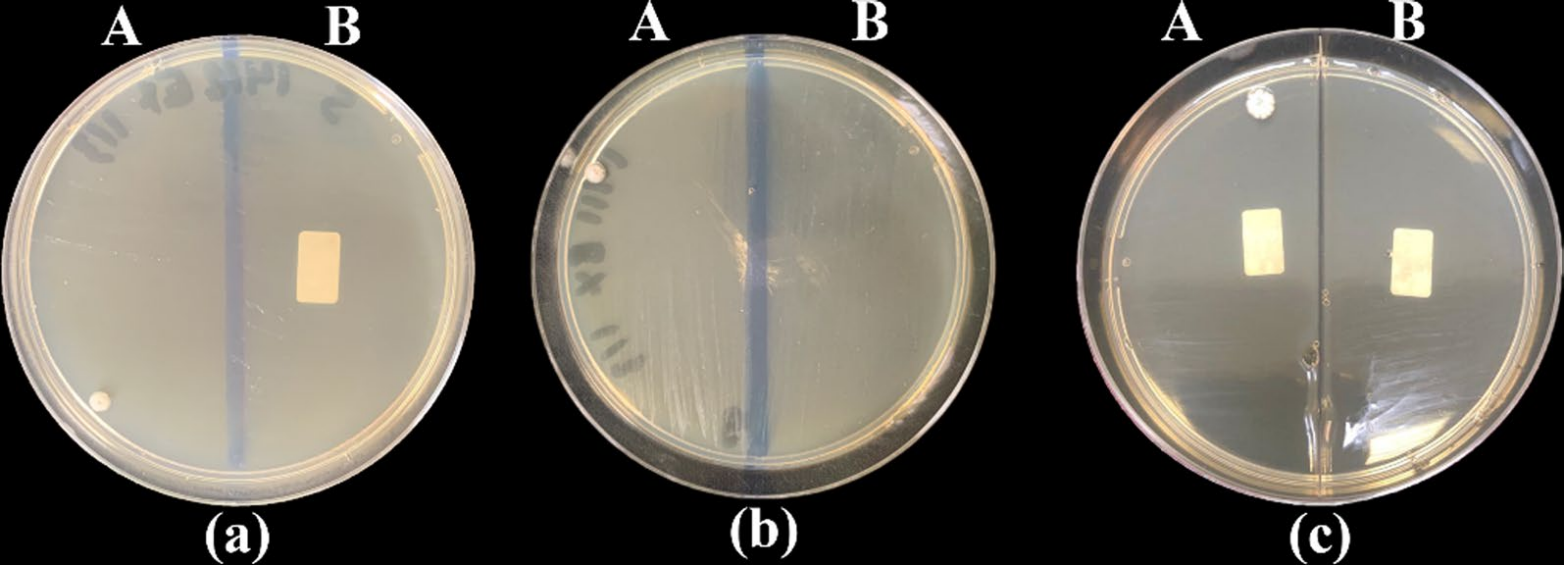
Culture results of the control group (A) and study group (B) on Sabouraud dextrose agar plates after one day (**a**), one week (**b**) and two weeks (**c**) from the production stage

**Fig. 7:**
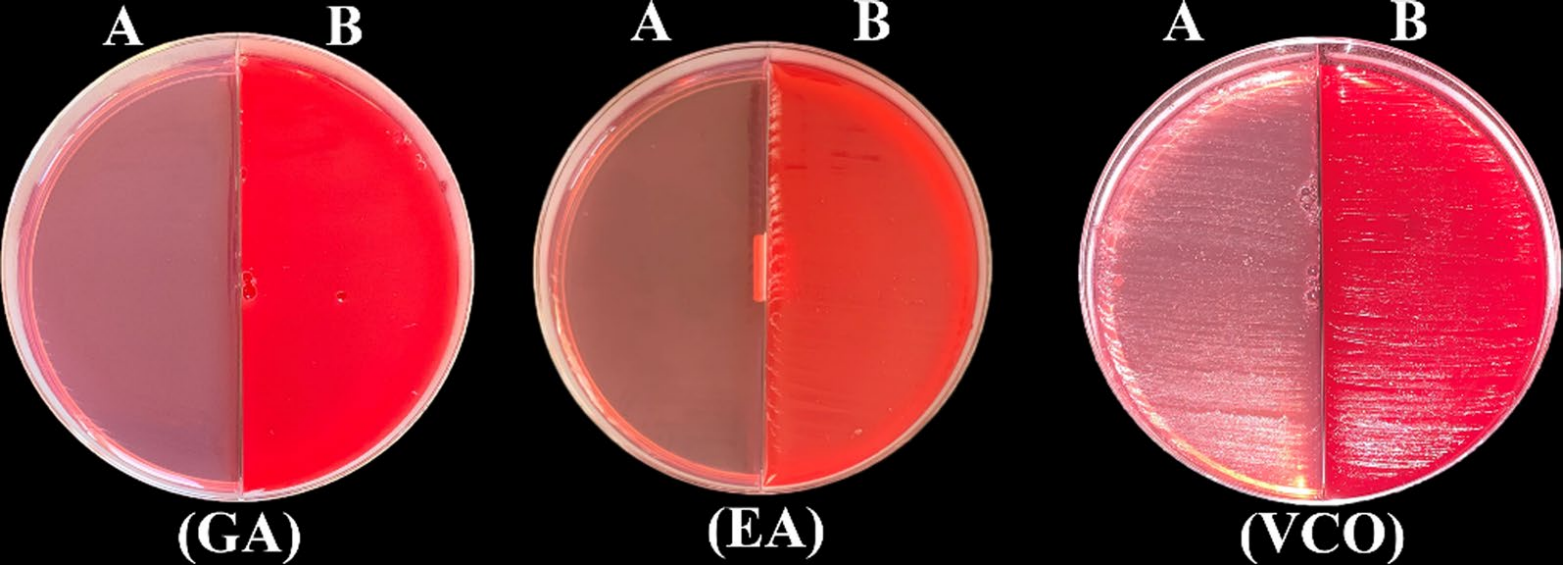
Culture results of the study groups GA, EA and VCO on MacConkey (**A**) and Blood (**B**) agar plates

No statistically significant difference was found between the second half of the three control groups untreated with any disinfectants with the mean microbial count after one day, one week, and two weeks from the production stage as the following;1.8, 2.8, and 3.6 CFU /plate respectively (*p* = 0.06) and the first half of the three study groups treated with the three disinfectants revealed no bacterial growth. However, a statistically significant difference was found between the three control and the three study groups (*p* < 0.001) (Table 4, Figs. 5, 6, 7, 11).

**Table 4:** Mean, standard deviation (SD) and the results of comparison of CFU counts between the three control and three study groups at different time-intervals from production stage of the surgical guides

Three types of culturing media (Agar plates)			Control Groups (VCO*, GA*, EA*)	Study Groups (VCO,GA,EA)	*p* (Between groups)
Blood agar	After 1 day	Mean ± SD	1.80 ± 0.79	0	< 0.001**
		Median (IQR)	2.00 (1.00, 2.25)	0	
	After 1 week	Mean ± SD	2.80 ± 1.32	0	< 0.001**
		Median (IQR)	3.00 (1.75, 4.00)	0	
	After 2 weeks	Mean ± SD	3.60 ± 2.27	0	< 0.001**
		Median (IQR)	3.50 (1.75, 5.25)	0	
MacConkey agar			0	0	N/A***
Sabouraud dextrose agar			0	0	N/A***
Percent change after 2 weeks		Mean ± SD	128.33 ± 169.98	0	< 0.001**
		Median (IQR)	83.33 (0.00, 225.00)	0	
P (Within group)			0.06	N/A***	

*IQR* interquartile range

*The second half of the three control groups

**Statistically significant *p* < 0.05

***Non applicable because the mean bacterial count = 0

## Discussion

**Fig. 8:**
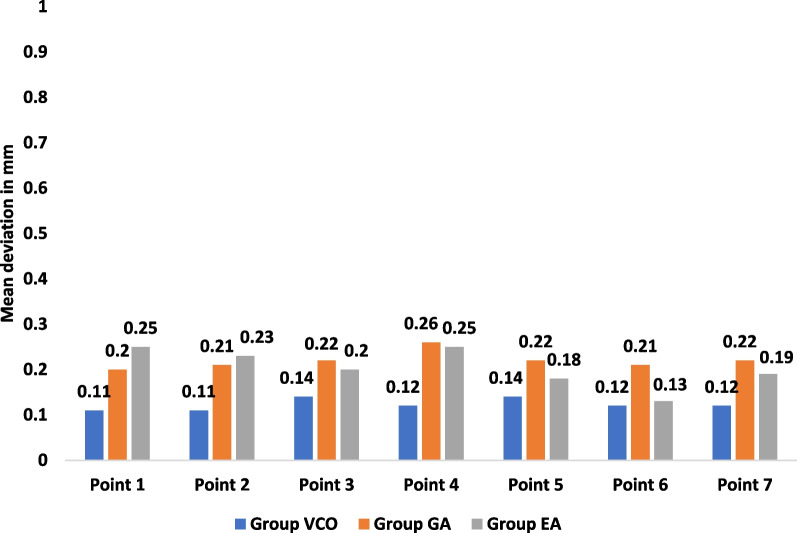
Morphological assessment of dimensional changes at seven points between the three study groups pre-and post-disinfection

**Fig. 9:**
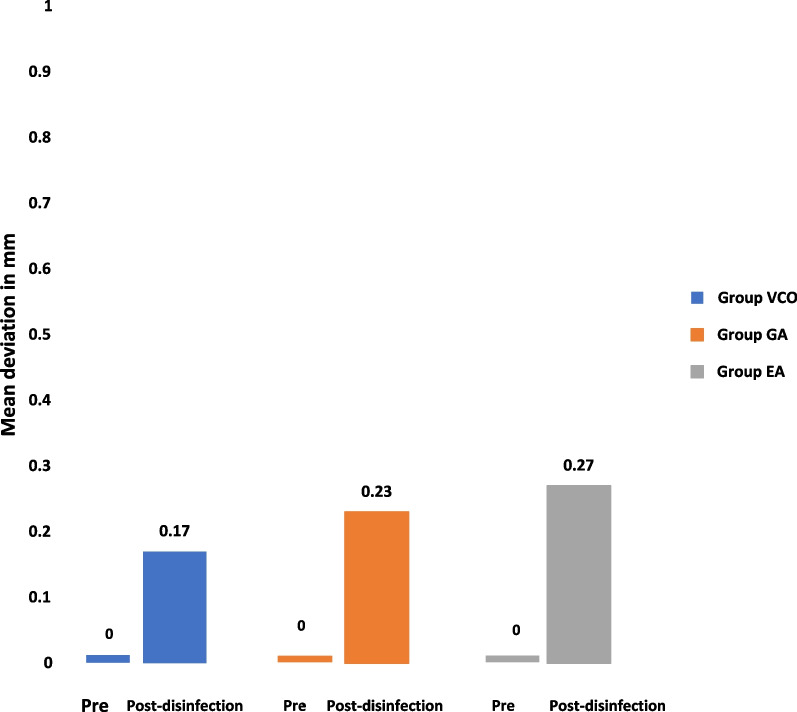
Volumetric assessment of dimensional changes at “point 8” between the three study groups pre- and post-disinfection

**Fig. 10:**
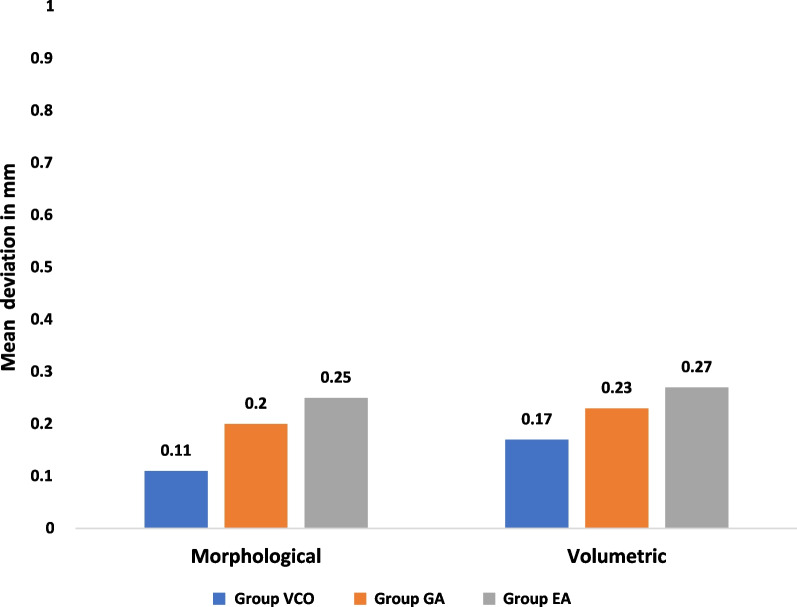
Average morphological and volumetric assessments of dimensional changes between the three study groups pre-and post- disinfection

**Fig. 11:**
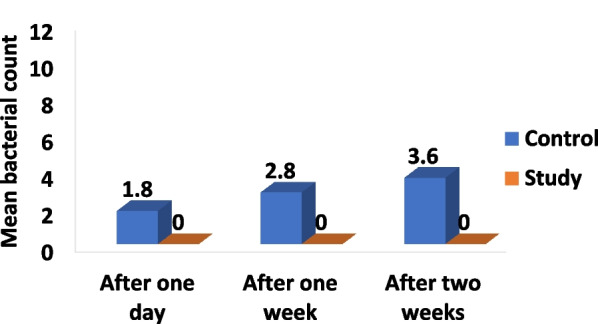
The mean bacterial count from the two halves of the surgical guides between the three control and three study groups at different interval times from the production stage of the surgical guides

In this study, the effect of disinfection on the morphological and volumetric dimensional changes of 3D printed surgical guides were evaluated and the antimicrobial effectiveness of the tested disinfectants used to decontaminate the tested surgical guides was assessed (Figs. 8, 9, 10, 11).

The null hypothesis was rejected regarding the morphological assessment as significant changes were found in the dimensions of the 3D printed surgical guides after immersion in 100% VCO, 2% GA, and 70% EA solutions, nonetheless, the null hypothesis was accepted regarding the volumetric assessment and the antimicrobial effectiveness of the three disinfectants showing 100% reduction in the microbial count of the microorganisms without causing any volumetric dimensional changes. Thereby, the results of the current study seem to confirm that there can be statistically significant variances in the morphological assessment between the three study groups. As expected, group VCO was more accurate showing the least morphological changes in comparison to group GA and EA, which were also submillimeter. This proven correlation is directly attributed to the potential of water absorption and the porous nature of the 3D printed resin material. This became a major concern due to the prolonged immersion in each respective disinfectant for 20 min, leading to the high possibility of absorption into the surgical guide material [38, 39]. Subsequently, the high viscosity of VCO (48.4–52.5 cP) could explain why it displayed minimal morphological changes, in comparison to GA and EA trials, which absorbed more easily into the surface area of the guides [40]. Therefore, as referenced in Fleischer et al. [38] suggested a reduction of porosity in the trial 3D-printed surface by employing appropriate printer settings and layer thickness which minimize the absorption of the disinfectant. This adjustment can improve sealing against fluid intake and avoid the risk of its contact with oral tissues as documented by Popescu et al. [12] Proving the dimensional changes of 0.2 mm appeared to be favorable, from the surgical point of view, and, further, did not influence the clinical use of the surgical guides [41, 42]. The present study showed significant differences in volumetric assessments between the three study groups in mean deviation of the x, y, and z axes at the center of the sleeve space, represented by point {8} the entry point of the first drill in various guided surgery protocols which affects the precise implant placement, where group GA and group EA represented higher volumetric changes than group VCO. Despite that, this variance was statistically insignificant when compared between the three study groups; still, group VCO showed minimal volumetric changes. When reviewing the microbiological results, there was a significant difference between the control and the study groups. However, it was demanding to pinpoint a specific justification for the negligible variance found between the three control groups. The rationale could be due to the prescribed antiseptic measurements. For instance, utilizing Isopropyl Alcohol as a disinfectant agent during post-processing coupled with the use of a sterile disk to cut the surgical guides into two halves and/or the use of sterile gloves when handling, could have limited the actual potential number of microorganisms recognized at varied time intervals between the three control groups. Thus, the clinical relevance of this study was disinfection using VCO may be considered as it was found to have no effect on the dimensions of surgical guides and with beneficial inhibitory actions on the microbial growth of the microorganisms.

Chepelev et al. [35] suggested that some forms of 3D printing were intrinsically sterile due to the high-temperature accumulation process associated with such thermoplastic materials. However, the practical applications involving post-processing, removal of residual support material, and transportation with multiple transfers could cause contamination with microorganisms. In addition to that, the surgical guides must be air-dried at room temperature in a digital lab environment, this would increase the possibility for bacterial contamination as well as promote the growth of microorganisms between the control groups. On the contrary, this finding was in opposition to Smith et al. [36] who conducted a study to estimate the microbial contamination of surgical guides before their intraoperative use with the ultimate outcome that surgical guides contained microorganisms before disinfection.

Interestingly, the results of the current study were consistent with Dewi et al. [43] that reported a significant change in the dimensions of alginate dental impressions when using VCO as a disinfectant in comparison to Sodium Hypochlorite and GA solutions, within the defined range, stated by the American Dental Association (ADA). Similar results were reinforced with Ósk Thorgeirsdóttir et al. [44] studying the effect of monocarpic acid as a disinfectant for dentures with strong antimicrobial activity against Candida when applied topically. Hence, the results presented in this study agreed with Mythri et al. [20] which tested a theory of the formation of sodium laurate, the main component of soap, where the interaction of lauric acid found in VCO with sodium hydroxide and bicarbonates found in saliva, had a cleansing effect, and decreased plaque formation in the oral cavity. Also, the presence of lauric acid inhibited the growth of *Staphylococcus aureus, Bacillus cereus, Salmonella typhimurium,* and *Escherichia coli.* Gayatri et al. [21] proposed an in vitro experiment that tested the antibacterial effects of VCO on the bacterial viability of *Actinomyces and Prevotella* species that caused tooth discoloration in children, and it was concluded that 100% VCO significantly reduced the viability of *Prevotella* species. This outcome supported the efficiency of 100% VCO employed in the current study. Furthermore, a study by Widianingrum et al. [22] showed that VCO could increase the potential of phagocytic immune cells; hence, it may be utilized as an alternative to antibiotics. In addition to that, Horas et al. [45] found that the topical effect of VCO on the palatal surgical wound during the palatoplasty procedure accelerated the wound healing, increased the number of fibroblast cells that appeared in the wound, and diminished the pain symptoms. Whereas, Thahir et al. [46] proved that VCO with its high lauric acid content accelerated the tissue healing and regeneration process in periodontal disease due to its ability to increase cellular metabolism, and collagen fibers density. Subsequently, Zicker et al. [47] investigated the anti-oxidant effect of VCO on bone osteopenia and microarchitecture in mice model and it was elucidated that mice supplemented with VCO not only, had a significantly greater bone volume and trabecular number but also, improved bone structure and prevented bone loss.

Moreover, the findings of the present study were comparable to the results of Sennhenn-Kirchner et al. [48] which recommended the pre-surgical use of 70% EA for 15–20 min; yet, the influence on the dimensions of the surgical guides was not investigated. On the other hand, in an in vitro study by Tallarico et al. [49], it was concluded that routine use of EA for 15 min before surgery, represented an effective procedure for disinfection with a high level of accuracy in the morphological characteristics of the tested surgical guides. Similarly, Akshaya et al. [50] compared the efficiency of decontamination of surgical instruments using 6% Sodium Hypochlorite and 2% GA. Though both chemical solutions are universally accepted by the CDC and ADA, 2% GA showed better results and more efficacy in decontamination than 6% Sodium Hypochlorite. Most recently, Matheus et al. [51] investigated the dimensional stability of the stereolithographic surgical guides after using 2% GA for 10 h as a chemical sterilant and it was proved to be a favorable choice of sterilization without changing the linear precision of the tested surgical guides. Török et al. [10] research was in opposition to the present efforts due to the implication of dissimilar 3D-printed material produced by Polyjet technology and a contrasting disinfectant (4% Gigasept), so the effect of the disinfection method was based only on the aspect of the tested material.

Moreover, in an era where infection control has taken the front stage as ever, evolving communicable pathogens and disinfecting agents have become a premier area of erudition and investigation. The sudden emergence, rapid spread, and continued mutation of Severe Acute Respiratory Syndrome Coronavirus 2 (SARS-COV-2) or COVID-19 have got worldwide attention towards the importance of disinfection protocols in the healthcare setting, and the population, as well [52]. Although the World Health Organization (WHO) recommended many chemical disinfectants that are readily accessible to fight the SARS-CoV-2 virus on surfaces, some of these disinfectants like EA, Isopropyl Alcohol, Hydrogen peroxide, and Hypochlorite solutions may cause risks to human health, as prolonged dermal absorption causes toxicity and frequent exposure can lead to an allergic condition of skin and eyes [53]. Exemplified this, Dayrit et al. [26] established a scientific motivation for the utilization of VCO as a potential adjuvant therapy for COVID-19 patients and a general prophylactic agent against various microbial infections.

Nevertheless, it is important to point out that the use of VCO as a disinfectant was not frequently addressed in dentistry, this is why its antimicrobial efficiency was compared with 2% GA and 70% EA solutions the commonly-used disinfectants in the dental field [10]. Even though the current study praised the antimicrobial effectiveness of the three disinfectants in eliminating 100% of the microorganisms found on the surgical guides, there were concerns about the serious adverse health effects documented among employees exposed to GA vapor such as asthma, rhinitis and, a chronic condition characterized by bronchial hyperresponsiveness, so these considerations had been viewed as a limitation for its use [13]. Besides that, Shi et al. [50] conducted that residual GA was a potential mutagen, which induced significant cytotoxic and mutagenic effects in mouse lymphoma cells. Although EA had substantial antimicrobial properties, it evaporated rapidly making extended exposure time difficult to achieve unless the items were immersed [13]. Subsequently, in an in vitro study, EA was unable to completely inactivate SARS-CoV-2 after 15 s of contact [53]. Research suggested that EA had no noticeable persistent residual activity when applied to the skin, however, the regrowth of bacteria occurred slowly after its use due to the sublethal effect that EA might have had on the residual bacteria [54]. Bondurant et al. [55] study had also shown that certain pathogen populations were becoming more tolerant to EA exposure and suggested the use of different antibacterial products in the clinical setting. Therefore, scientists must explore novel methods that have been present in our ecosystem for centuries such as herbal and/or organic remedies. Thus, this designed in vitro study addressed VCO as a promising potent disinfectant with a unique source of natural nontoxic products, easily applicable, affordable cost-effective, virtually risk-free, environmentally friendly, and with enormous potential benefits.

## Limitations

However, the current study is not without limitations. Firstly, the measurements were limited to the deviations at the center of the sleeves in xyz axes pre- and post- disinfection and the accuracy of guided implant surgery depends not only on the correct point of entry but also, on the angulation during drilling. However, such angulation could be affected in vivo by other confounding factors such as the guided protocol that is followed and the type of support teeth, bone, or mucosa. Finally, the effect of the tested disinfectants was based only on the aspect of material testing. Therefore, further studies are required to be conducted using different types of surgical guide materials to obtain final conclusions.

## Conclusions

Based on the findings of this in vitro study, the following conclusions were drawn:

1. Disinfection using 100% VCO for 20 min had a minimal morphological effect on the dimensional accuracy of the tested surgical guides that was clinically acceptable.
2. Disinfection using 100% VCO for 20 min proved a lack of volumetric dimensional changes of the tested surgical guides.
3. The antimicrobial effectiveness of 100% VCO was comparable and equivalent to 2% GA and 70% EA with enormous potential benefits and exceptional outcomes**.**

### Recommendations

1. Future research on the volumetric measurement to investigate the deviations between the angulation plane of the sleeves and the angulation plane of the inserted implants in vivo when a fully guided protocol is to be followed on dentate patients.
2. Further investigations should take into account the characterization of bacterial morphotypes present on the guide surfaces before and after the antimicrobial treatment.
3. In vivo studies, VCO is highly recommended to evaluate its beneficial effect on surgical wound healing after implant placement and to determine its effect on bone loss prevention which affects the overall success of implant procedure.
4. There is a dire need for additional clinical trials which can investigate the effects of monolaurin on oral microorganisms and assess the effect of VCO pulling therapy which can be used as a potent mouthwash before implant surgery.
5. Further clinical studies must be conducted to investigate the impact of VCO as a surgical site disinfectant taking into consideration its use as a decontaminating agent that lacks harmful residues, provides an aseptic operating field, and prevent contamination during the implant procedure

## Supplementary Information

**Additional file 1.** Study data.

## Data Availability

All the data generated or analyzed during this study are included in this published article and its Additional file 1.

## Abbreviations

CDC: Center for disease control and prevention
VCO: Virgin coconut oil
GA: Glutaraldehyde
EA: Ethyl alcohol
3D: Three-dimensional
CBCT: Cone-beam computed tomography
DICOM: Digital imaging and communications in medicine
ANOVA: Analysis of variance
SD: Standard deviation
RMS: Root mean square
CFU: Colony-forming units
µl: Microliter
ADA: American Dental Association
WHO: World Health Organization
SARS-COV-2: Severe acute respiratory syndrome coronavirus
