# Supplementary material for: The microbiological effect of virgin coconut oil on the morphological and volumetric dimensional changes of 3D printed surgical guides (in vitro study)
Source: BMC Oral Health. 2022 Dec 23;22:636. doi: 10.1186/s12903-022-02671-8 (PMC9786529; doi:10.1186/s12903-022-02671-8)
Supplement: Supplementary file 1 — Additional file 1. Study data. [file 12903_2022_2671_MOESM1_ESM.zip › additional file/Graphs.docx]

**Graph** 1**.** Volumetric assessment of dimensional changes between the three study groups before and after disinfection.

**Graph 2.** Mean bacterial count between the three control and three study groups at different time-intervals from production stage of the surgical guides**.**
