# Supplementary figures and images for: The microbiological effect of virgin coconut oil on the morphological and volumetric dimensional changes of 3D printed surgical guides (in vitro study)
Source: BMC Oral Health. 2022 Dec 23;22:636. doi: 10.1186/s12903-022-02671-8 (PMC9786529; doi:10.1186/s12903-022-02671-8)

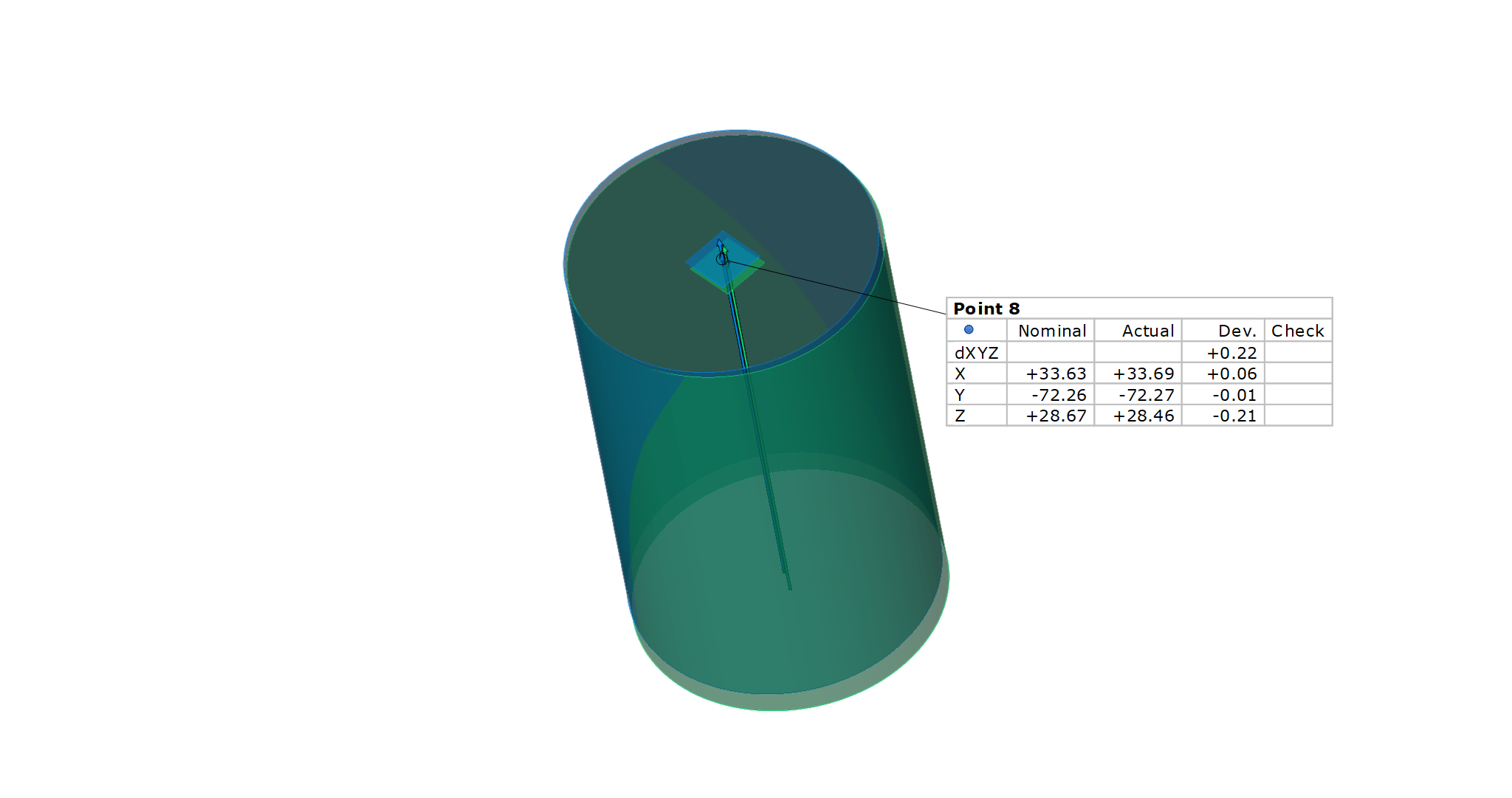

Supplement: Supplementary file 1 — Additional file 1. Study data. [file 12903_2022_2671_MOESM1_ESM.zip › additional file/Dimensional Changes Reports/Volume Comparison G1S1-G2S1.png]

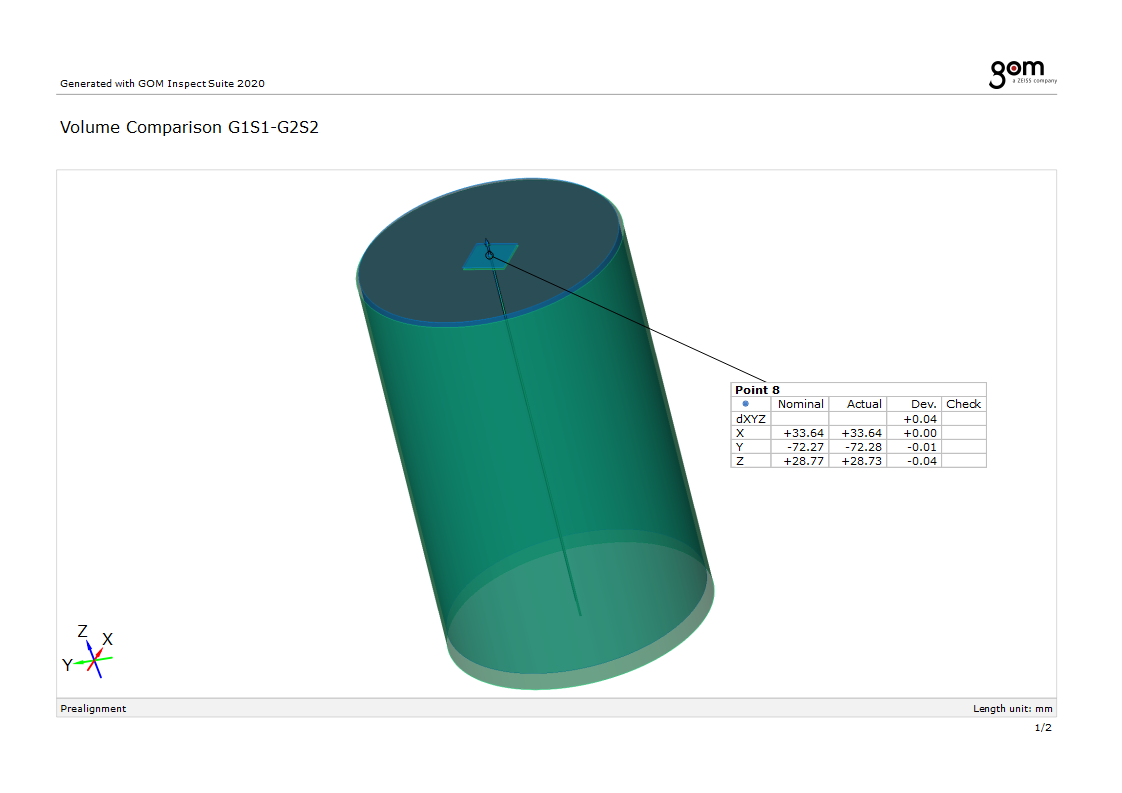

Supplement: Supplementary file 1 — Additional file 1. Study data. [file 12903_2022_2671_MOESM1_ESM.zip › additional file/Dimensional Changes Reports/Volume Comparison G1S1-G2S2.png]

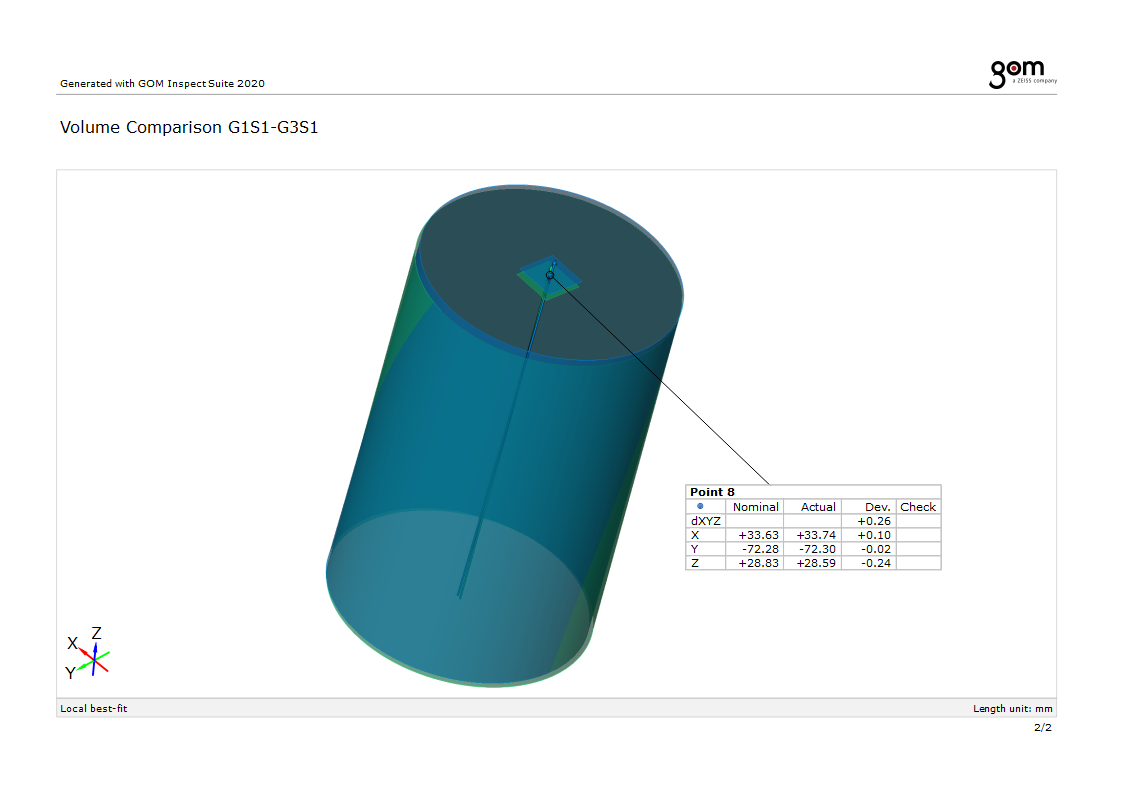

Supplement: Supplementary file 1 — Additional file 1. Study data. [file 12903_2022_2671_MOESM1_ESM.zip › additional file/Dimensional Changes Reports/Volume Comparison G1S1-G3S1.png]

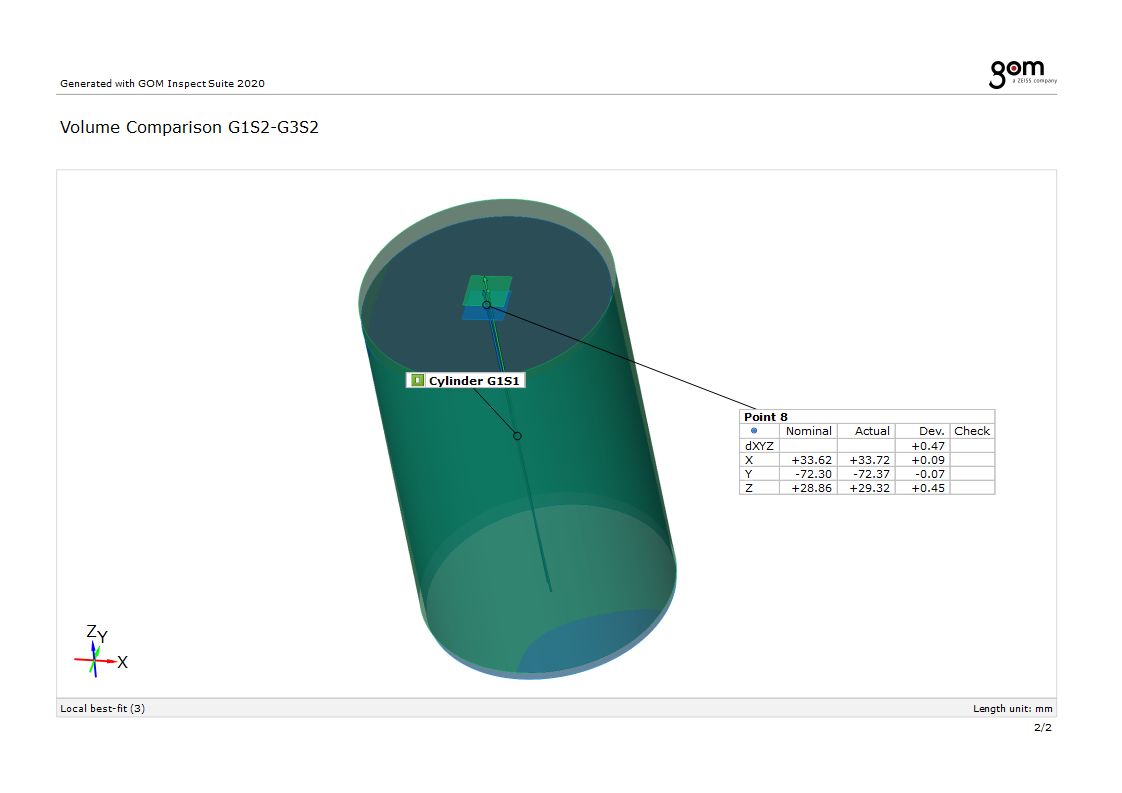

Supplement: Supplementary file 1 — Additional file 1. Study data. [file 12903_2022_2671_MOESM1_ESM.zip › additional file/Dimensional Changes Reports/Volume Comparison G1S2-G3S2.png]

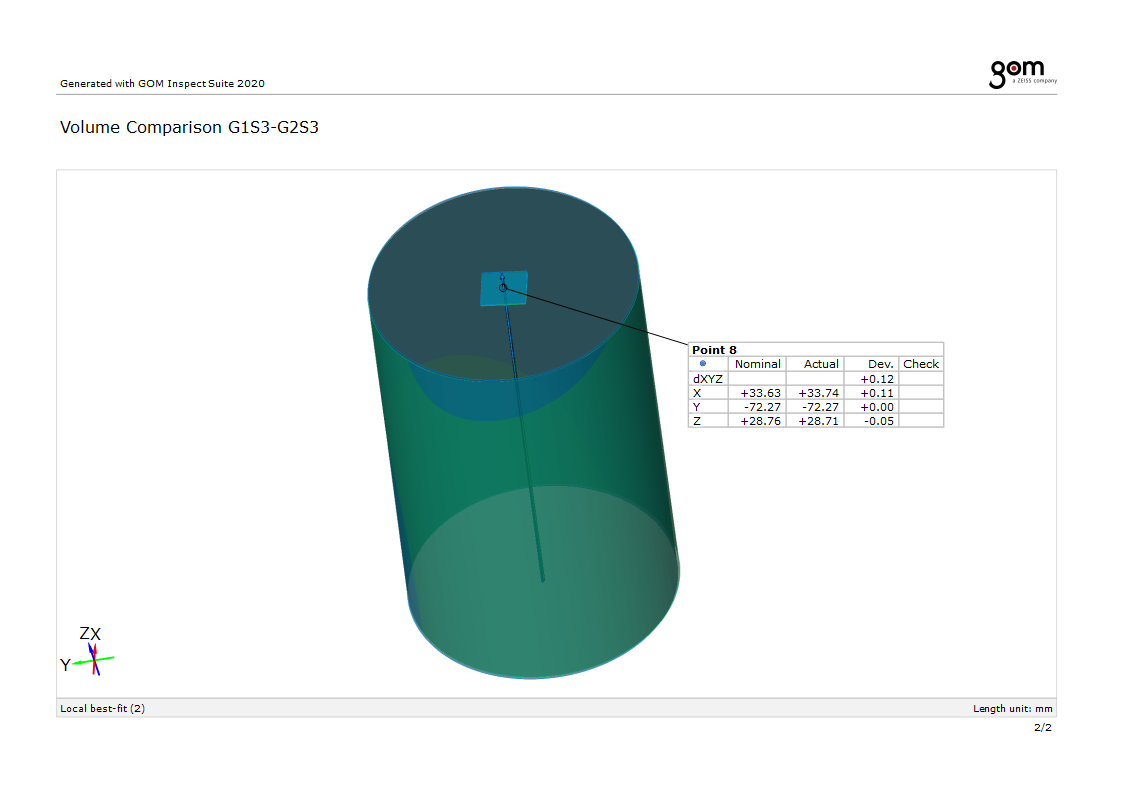

Supplement: Supplementary file 1 — Additional file 1. Study data. [file 12903_2022_2671_MOESM1_ESM.zip › additional file/Dimensional Changes Reports/Volume Comparison G1S3-G2S3.png]

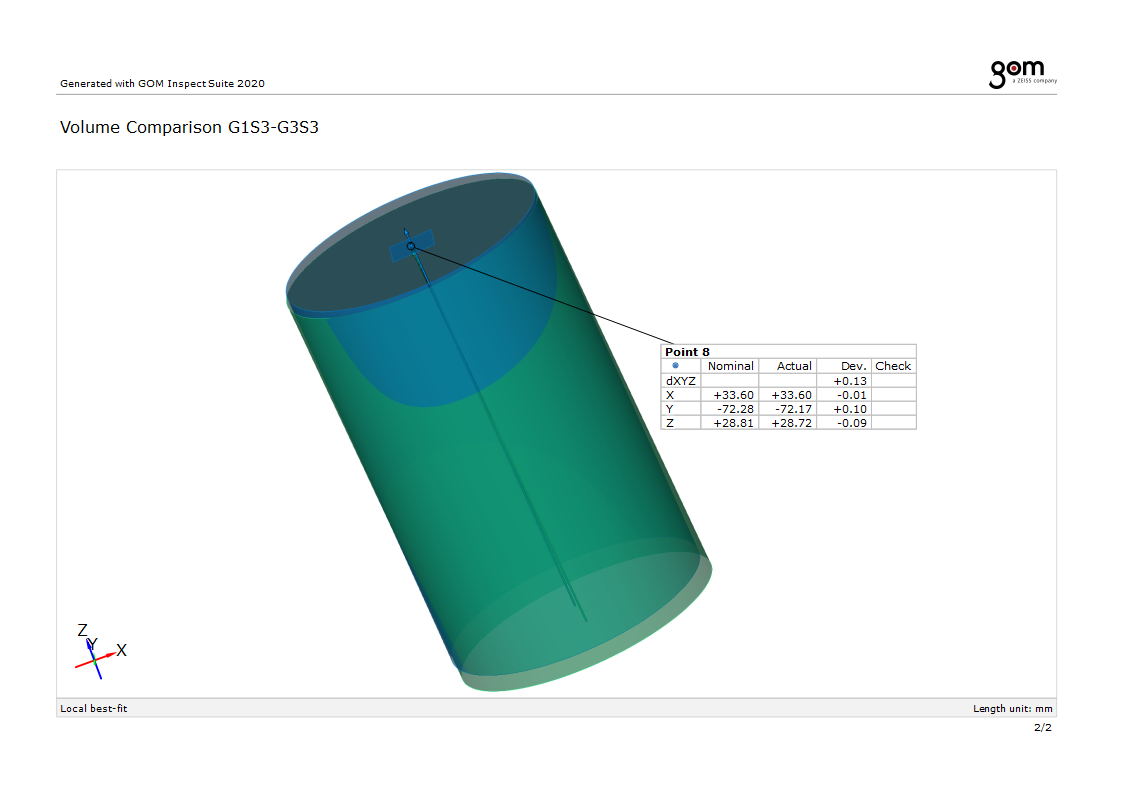

Supplement: Supplementary file 1 — Additional file 1. Study data. [file 12903_2022_2671_MOESM1_ESM.zip › additional file/Dimensional Changes Reports/Volume Comparison G1S3-G3S3.png]

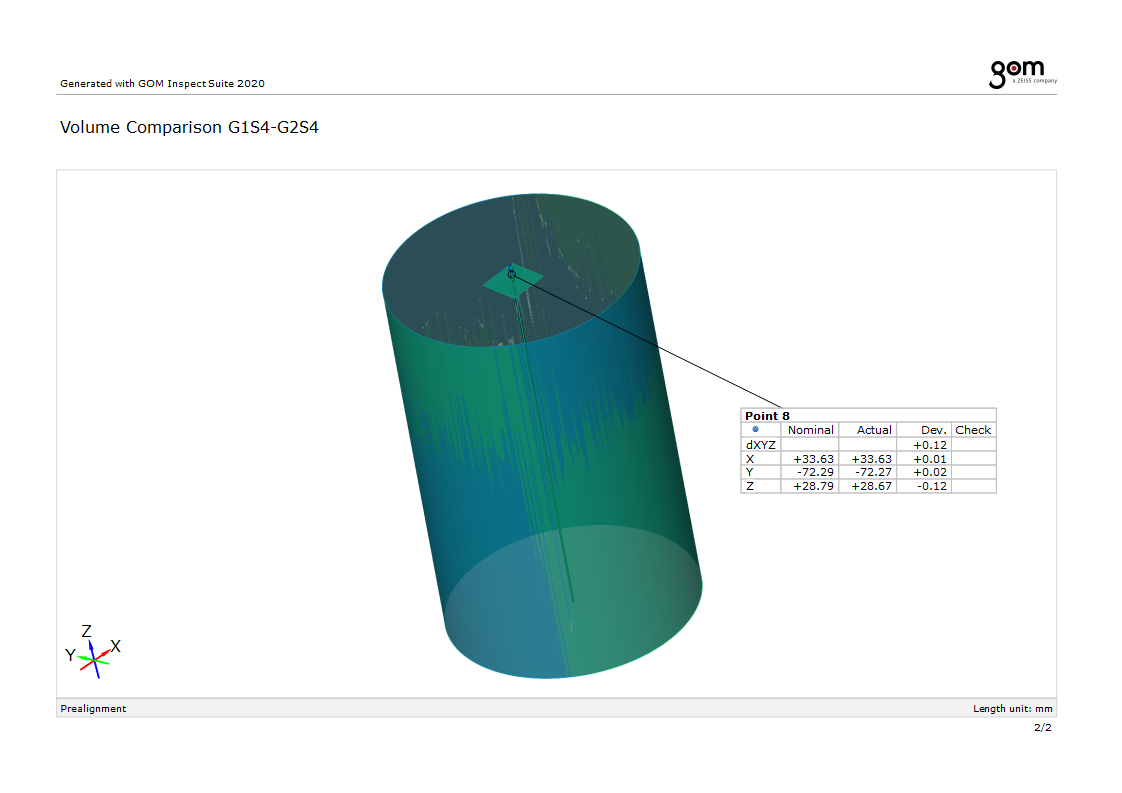

Supplement: Supplementary file 1 — Additional file 1. Study data. [file 12903_2022_2671_MOESM1_ESM.zip › additional file/Dimensional Changes Reports/Volume Comparison G1S4-G2S4.png]

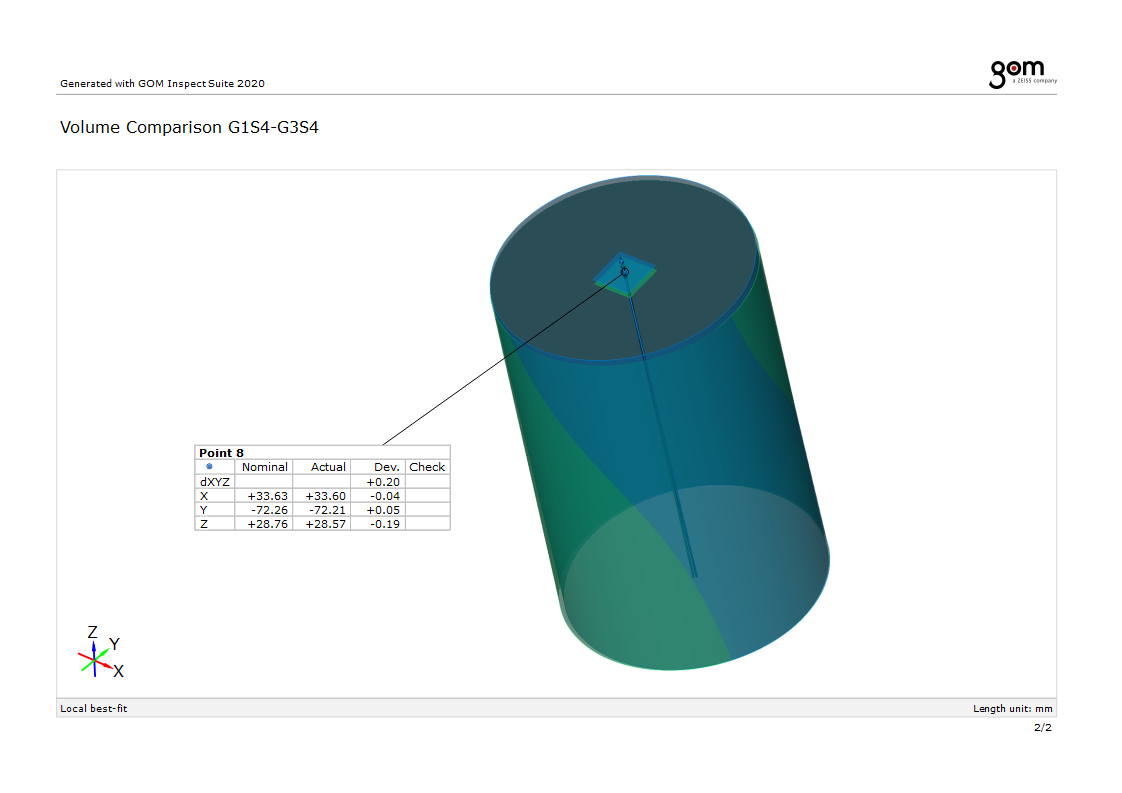

Supplement: Supplementary file 1 — Additional file 1. Study data. [file 12903_2022_2671_MOESM1_ESM.zip › additional file/Dimensional Changes Reports/Volume Comparison G1S4-G3S4.png]

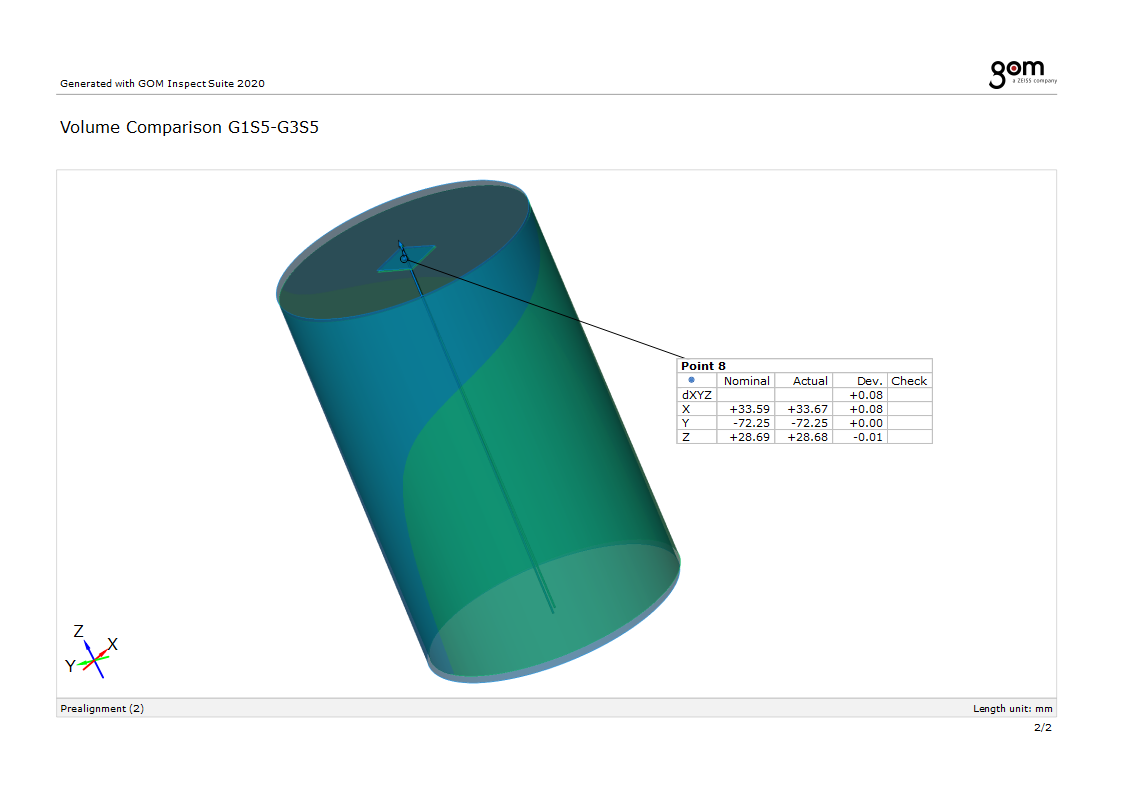

Supplement: Supplementary file 1 — Additional file 1. Study data. [file 12903_2022_2671_MOESM1_ESM.zip › additional file/Dimensional Changes Reports/Volume Comparison G1S5-G2S5.png]

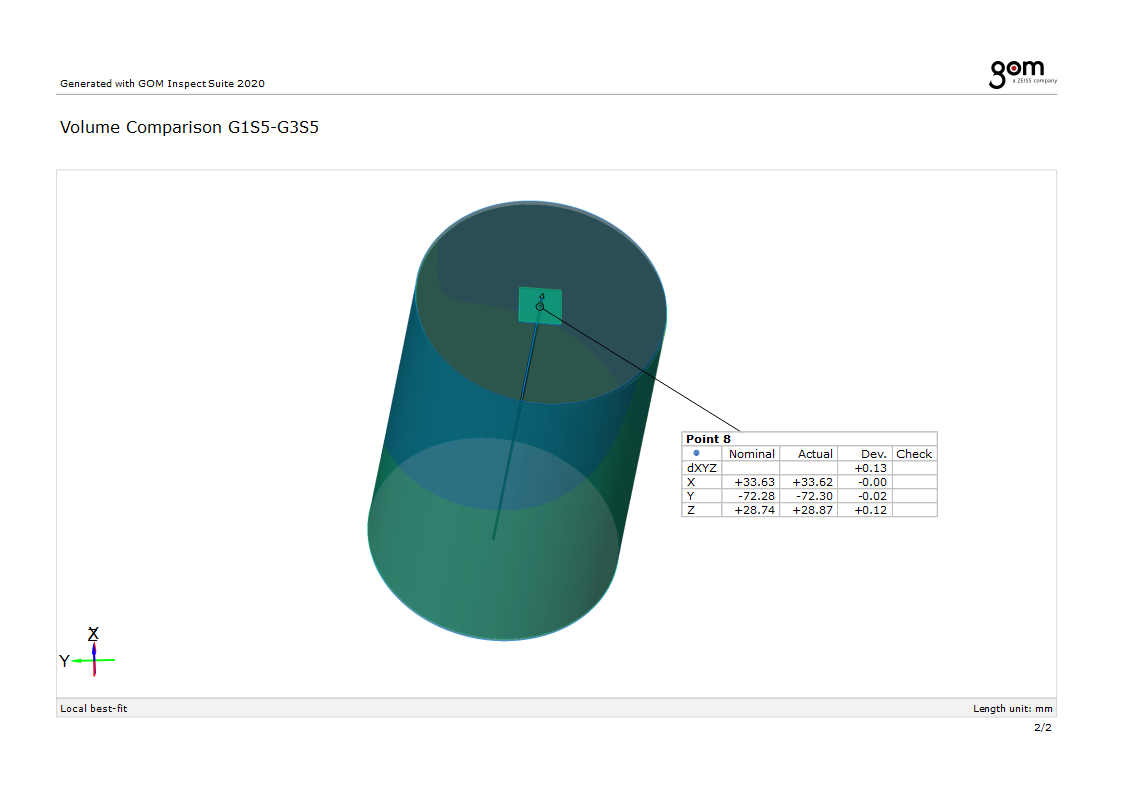

Supplement: Supplementary file 1 — Additional file 1. Study data. [file 12903_2022_2671_MOESM1_ESM.zip › additional file/Dimensional Changes Reports/Volume Comparison G1S5-G3S5.png]

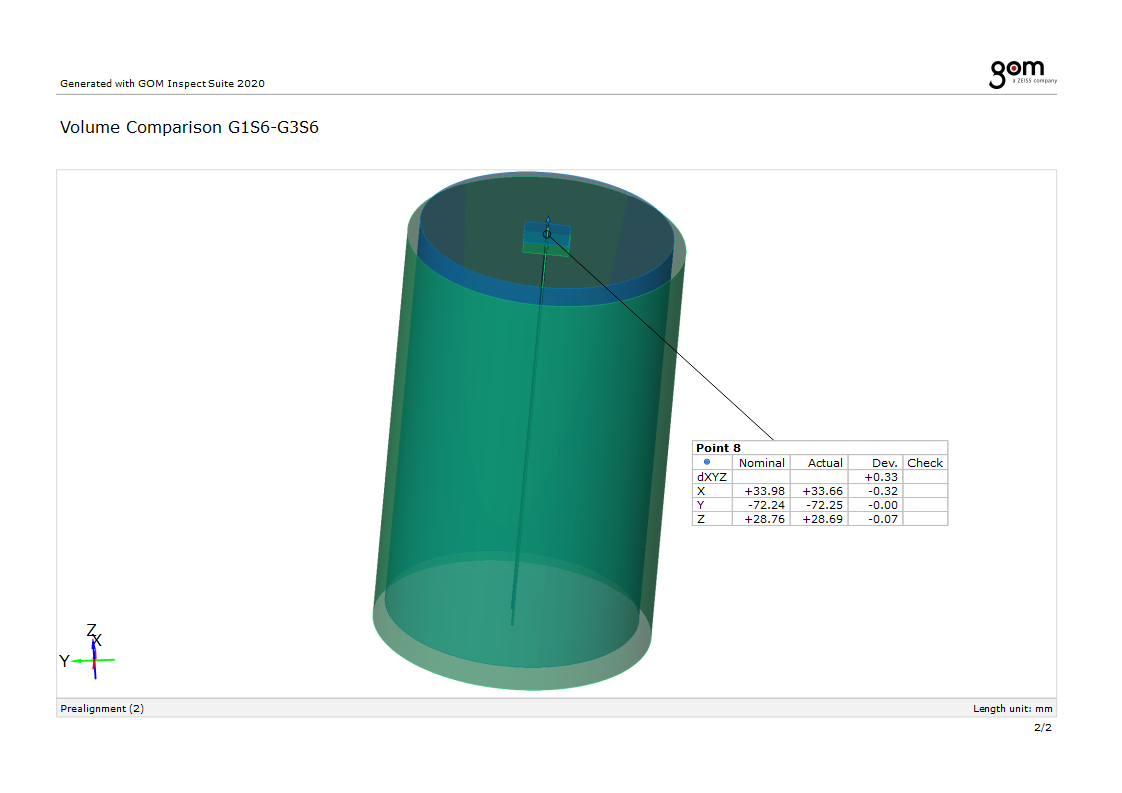

Supplement: Supplementary file 1 — Additional file 1. Study data. [file 12903_2022_2671_MOESM1_ESM.zip › additional file/Dimensional Changes Reports/Volume Comparison G1S6-G2S6.png]

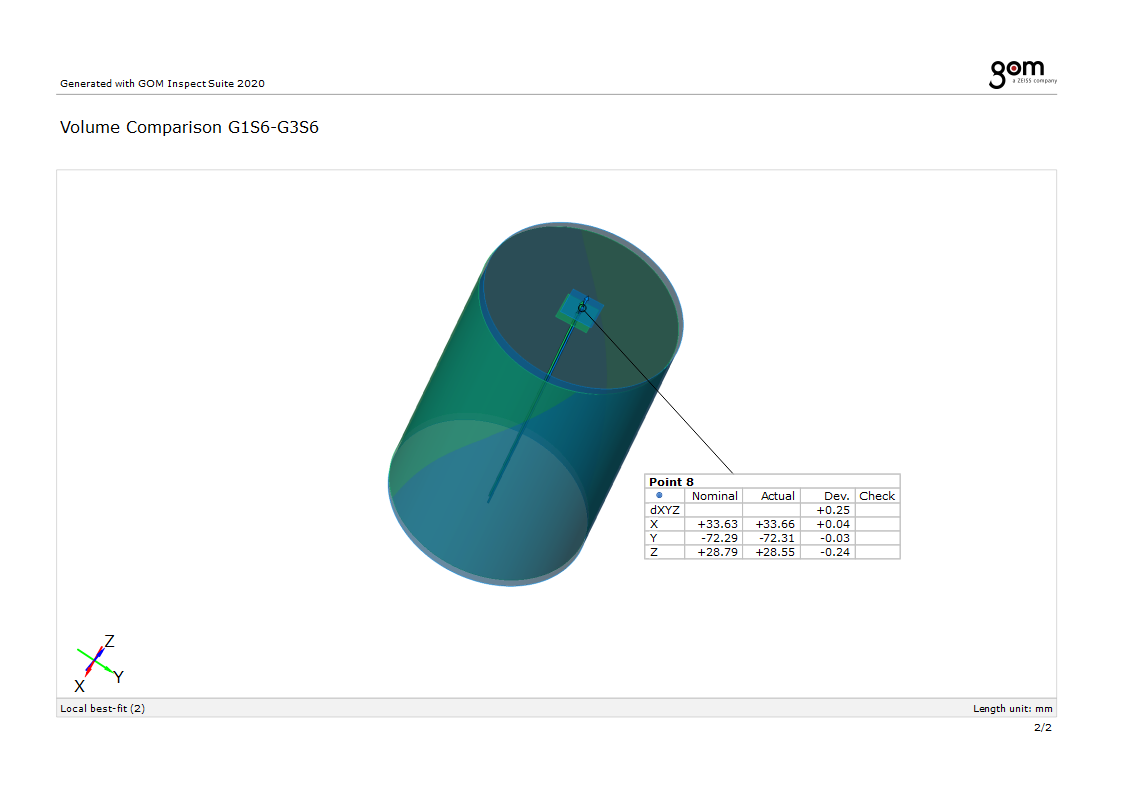

Supplement: Supplementary file 1 — Additional file 1. Study data. [file 12903_2022_2671_MOESM1_ESM.zip › additional file/Dimensional Changes Reports/Volume Comparison G1S6-G3S6.png]

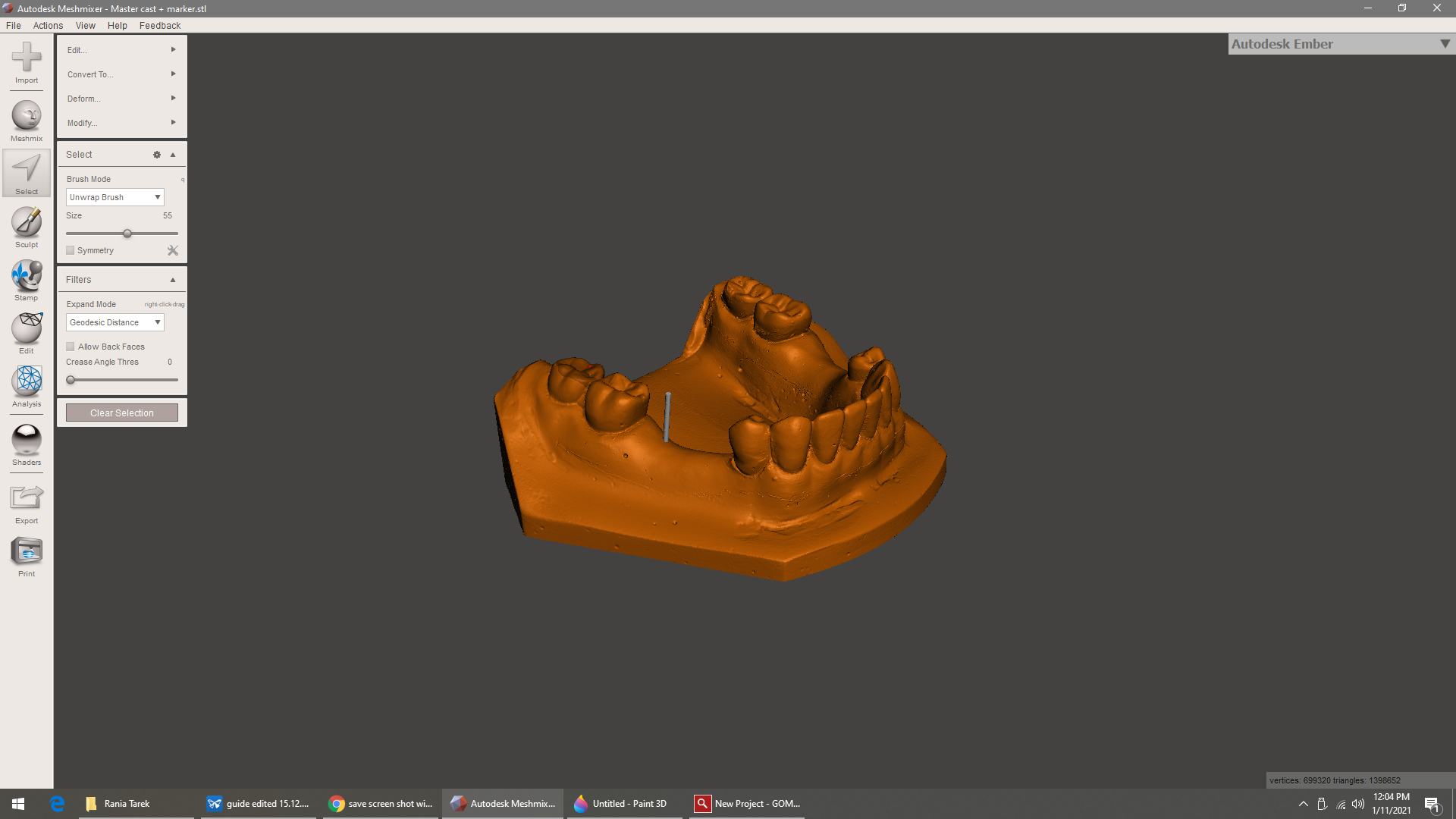

Supplement: Supplementary file 1 — Additional file 1. Study data. [file 12903_2022_2671_MOESM1_ESM.zip › additional file/Printing Process/Screenshot (1).png]

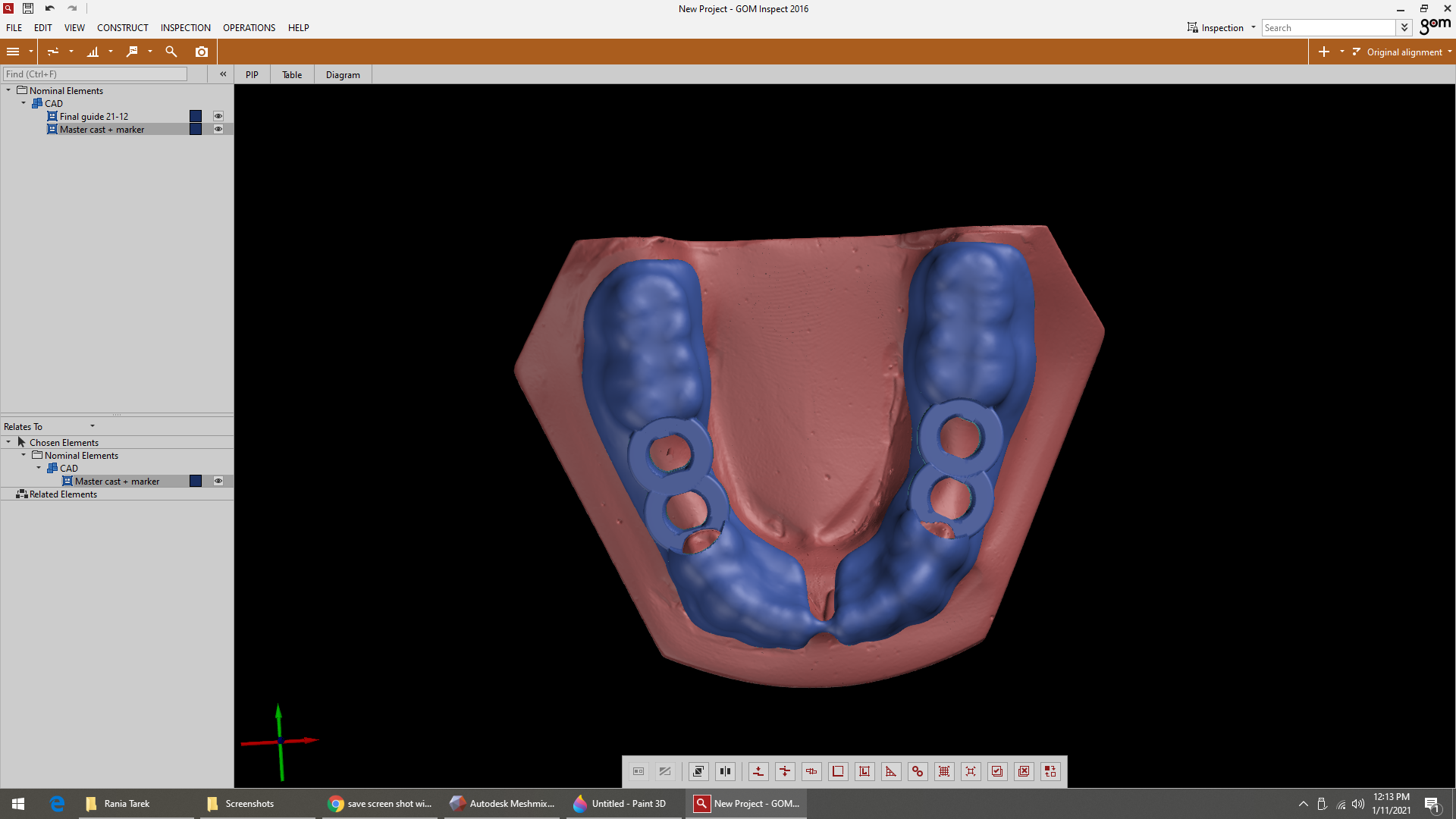

Supplement: Supplementary file 1 — Additional file 1. Study data. [file 12903_2022_2671_MOESM1_ESM.zip › additional file/Printing Process/Screenshot (10).png]

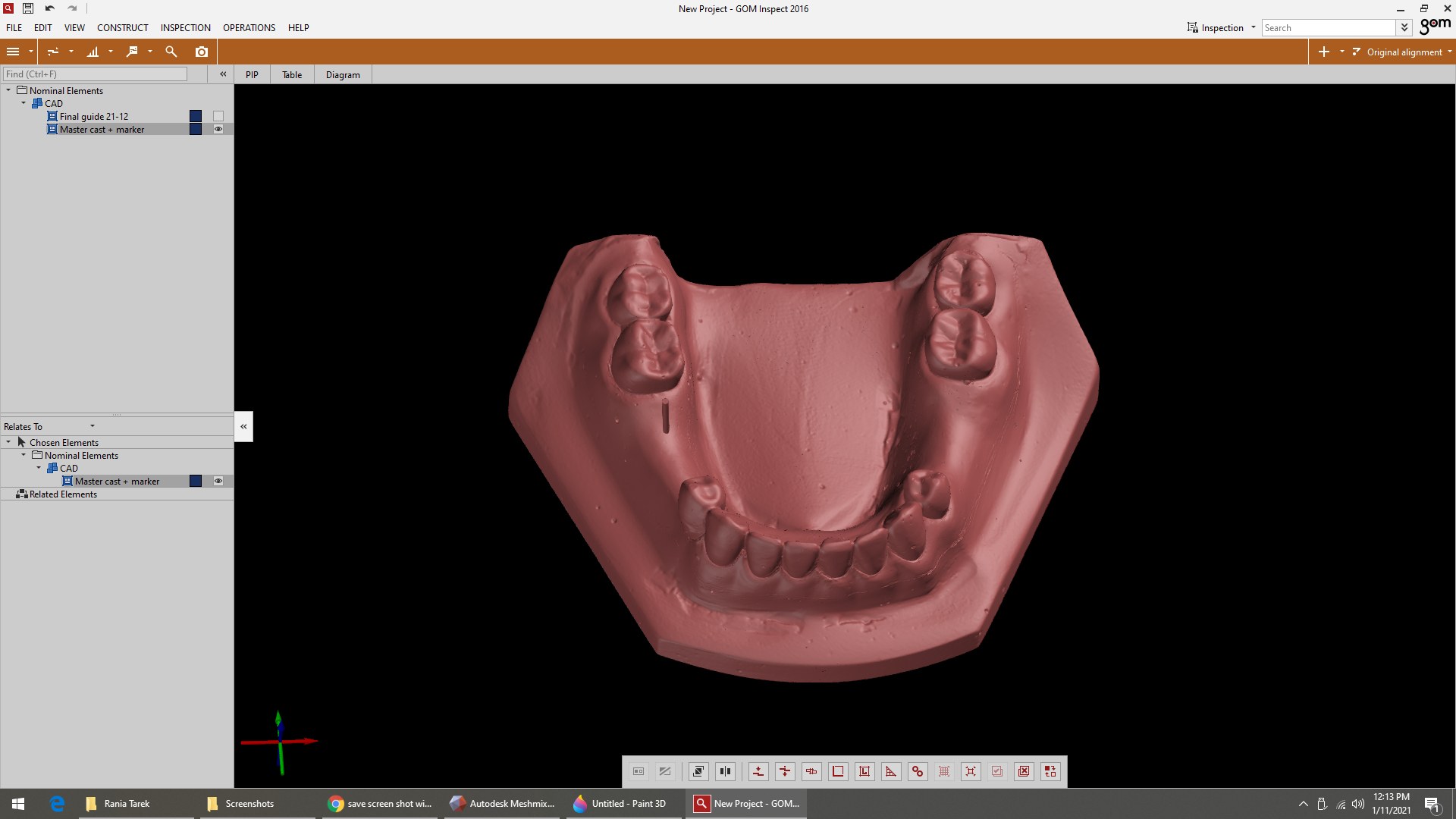

Supplement: Supplementary file 1 — Additional file 1. Study data. [file 12903_2022_2671_MOESM1_ESM.zip › additional file/Printing Process/Screenshot (11).png]

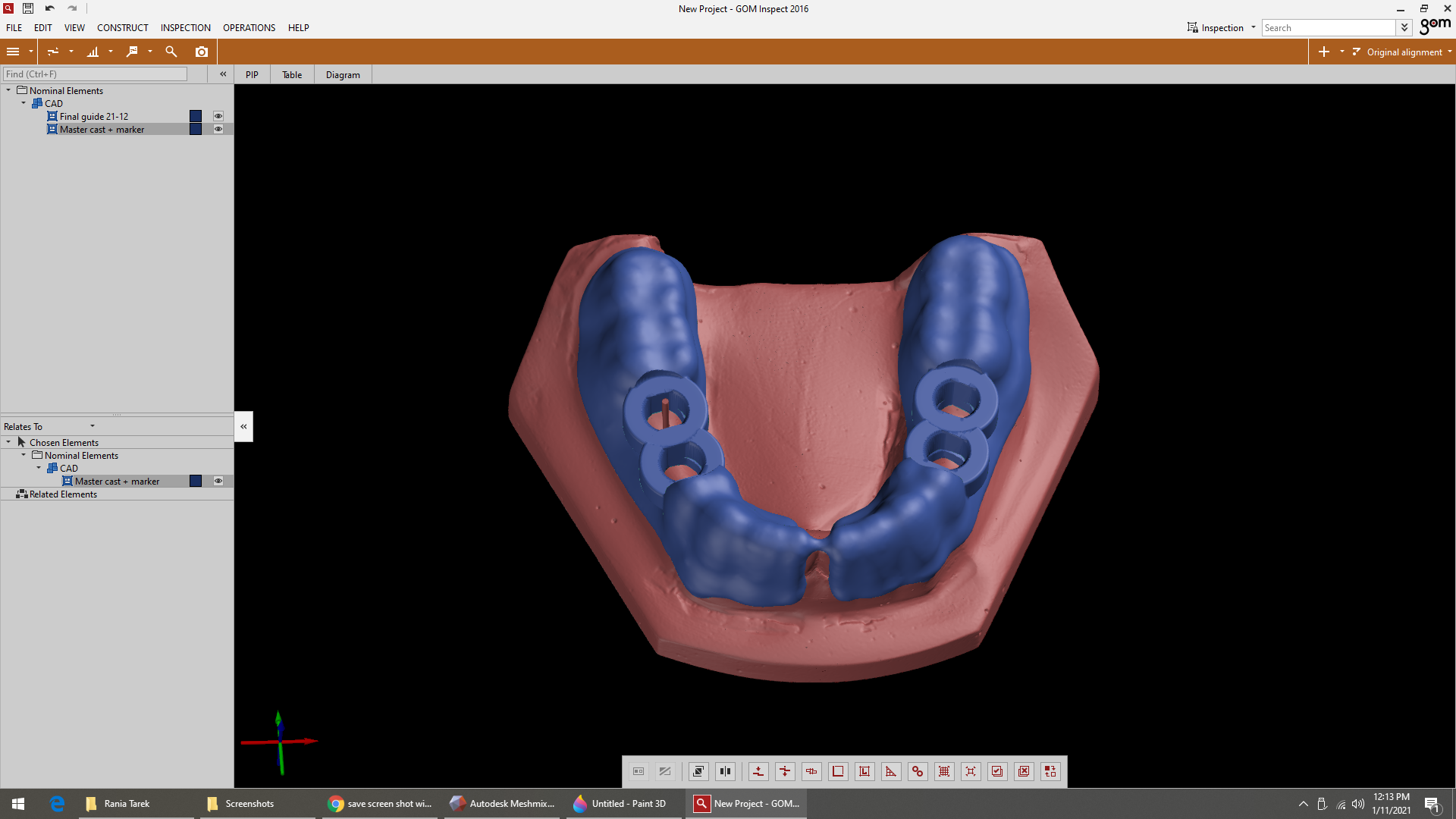

Supplement: Supplementary file 1 — Additional file 1. Study data. [file 12903_2022_2671_MOESM1_ESM.zip › additional file/Printing Process/Screenshot (12).png]

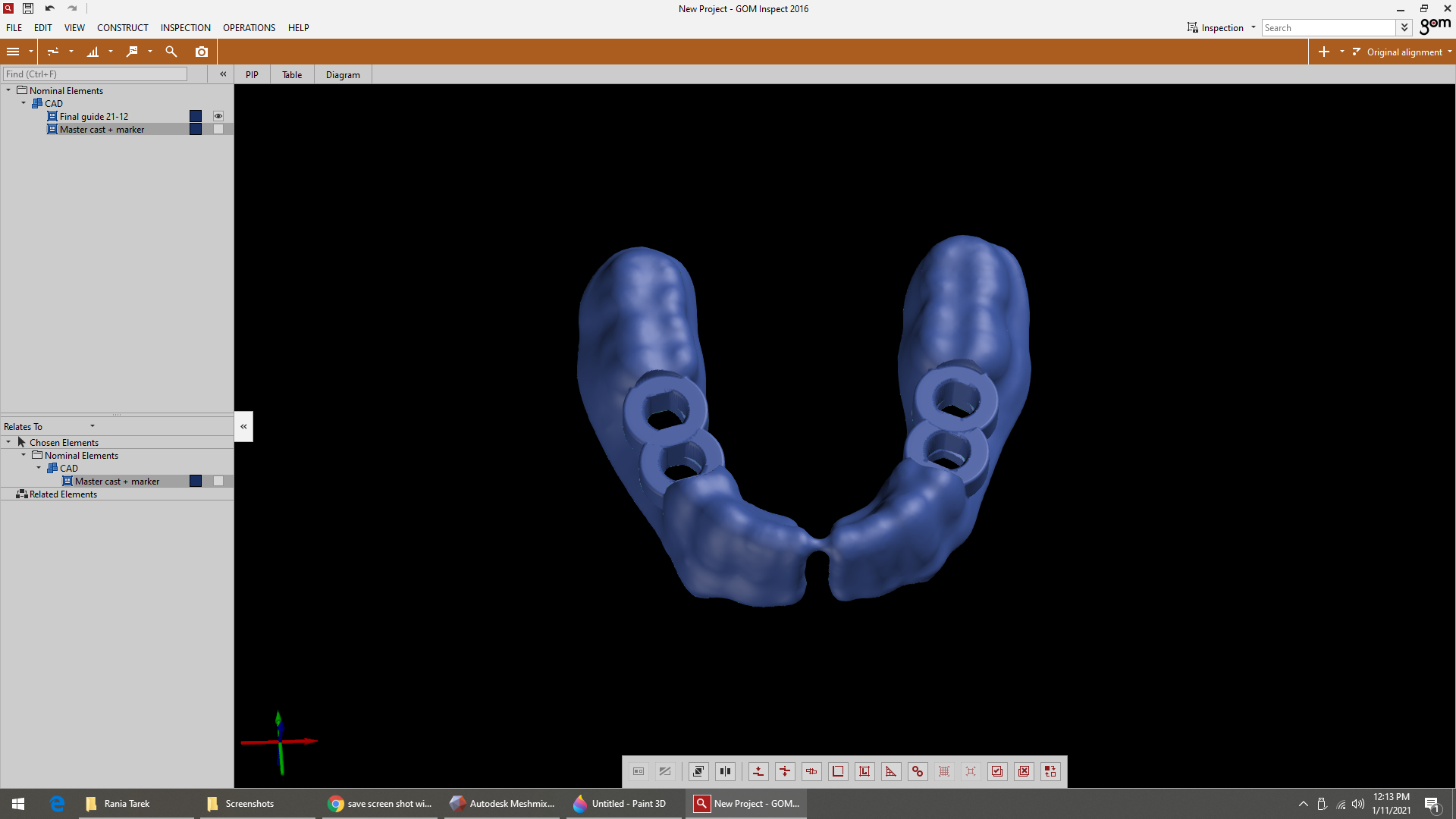

Supplement: Supplementary file 1 — Additional file 1. Study data. [file 12903_2022_2671_MOESM1_ESM.zip › additional file/Printing Process/Screenshot (13).png]

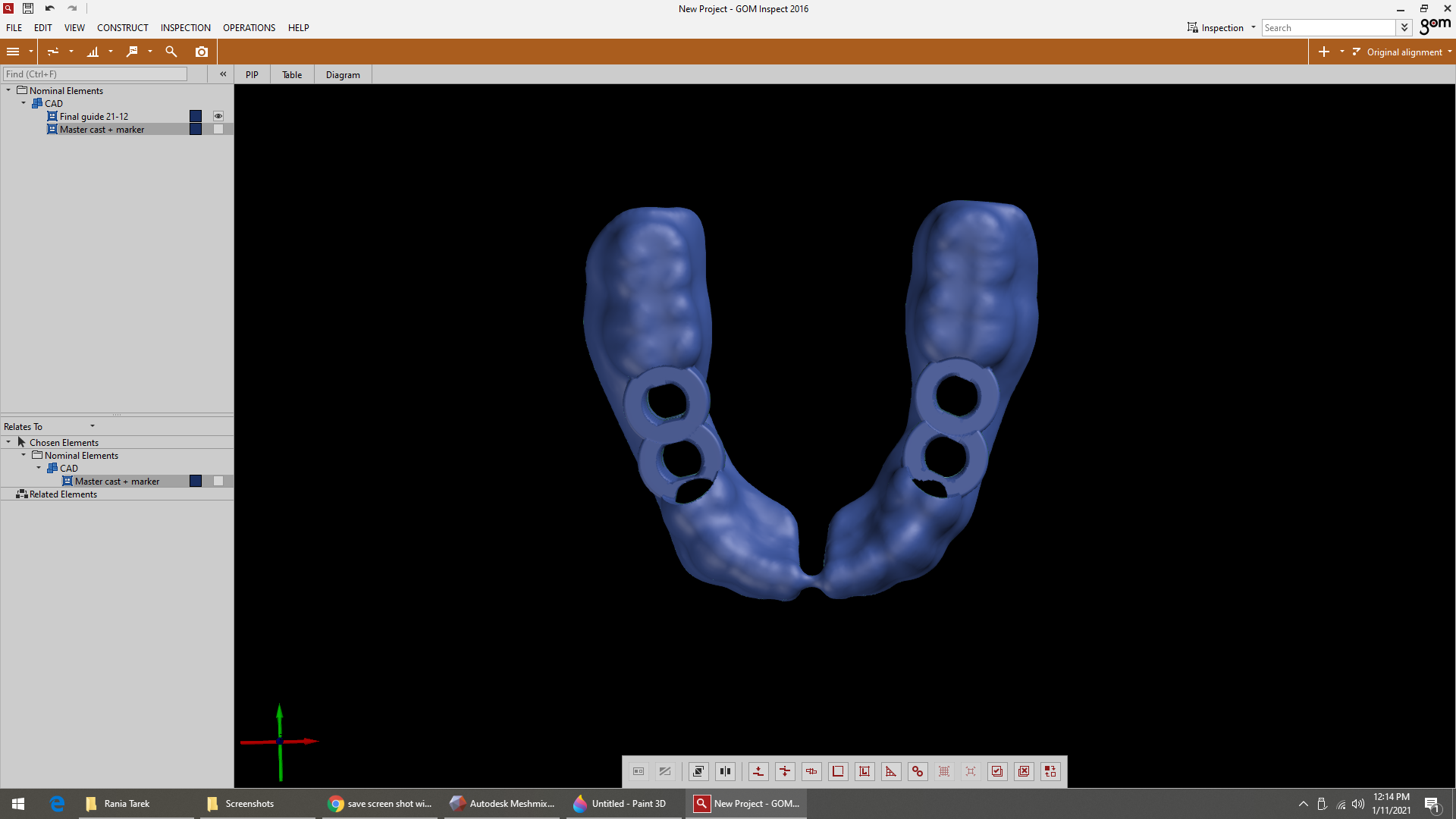

Supplement: Supplementary file 1 — Additional file 1. Study data. [file 12903_2022_2671_MOESM1_ESM.zip › additional file/Printing Process/Screenshot (14).png]

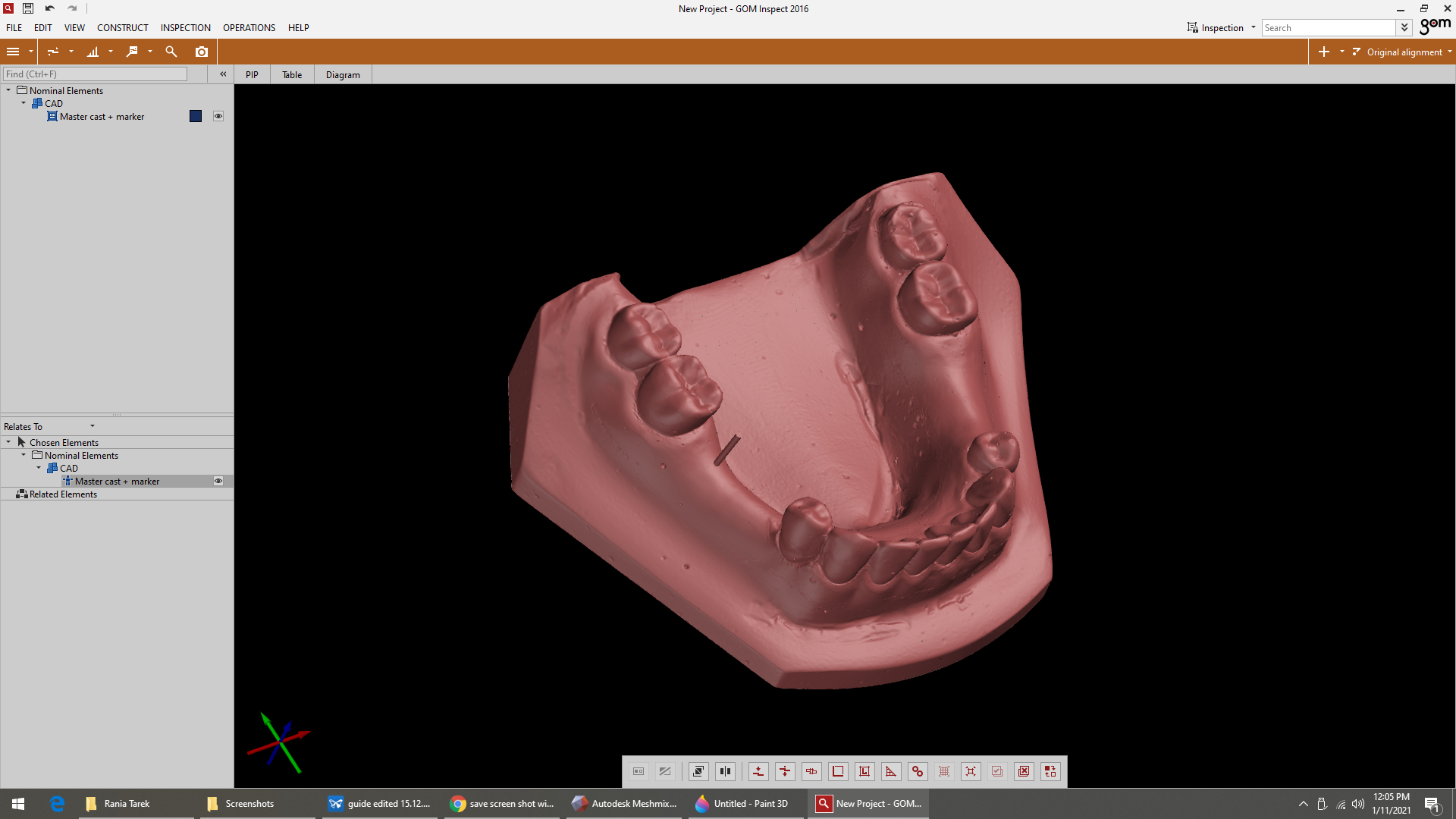

Supplement: Supplementary file 1 — Additional file 1. Study data. [file 12903_2022_2671_MOESM1_ESM.zip › additional file/Printing Process/Screenshot (2).png]

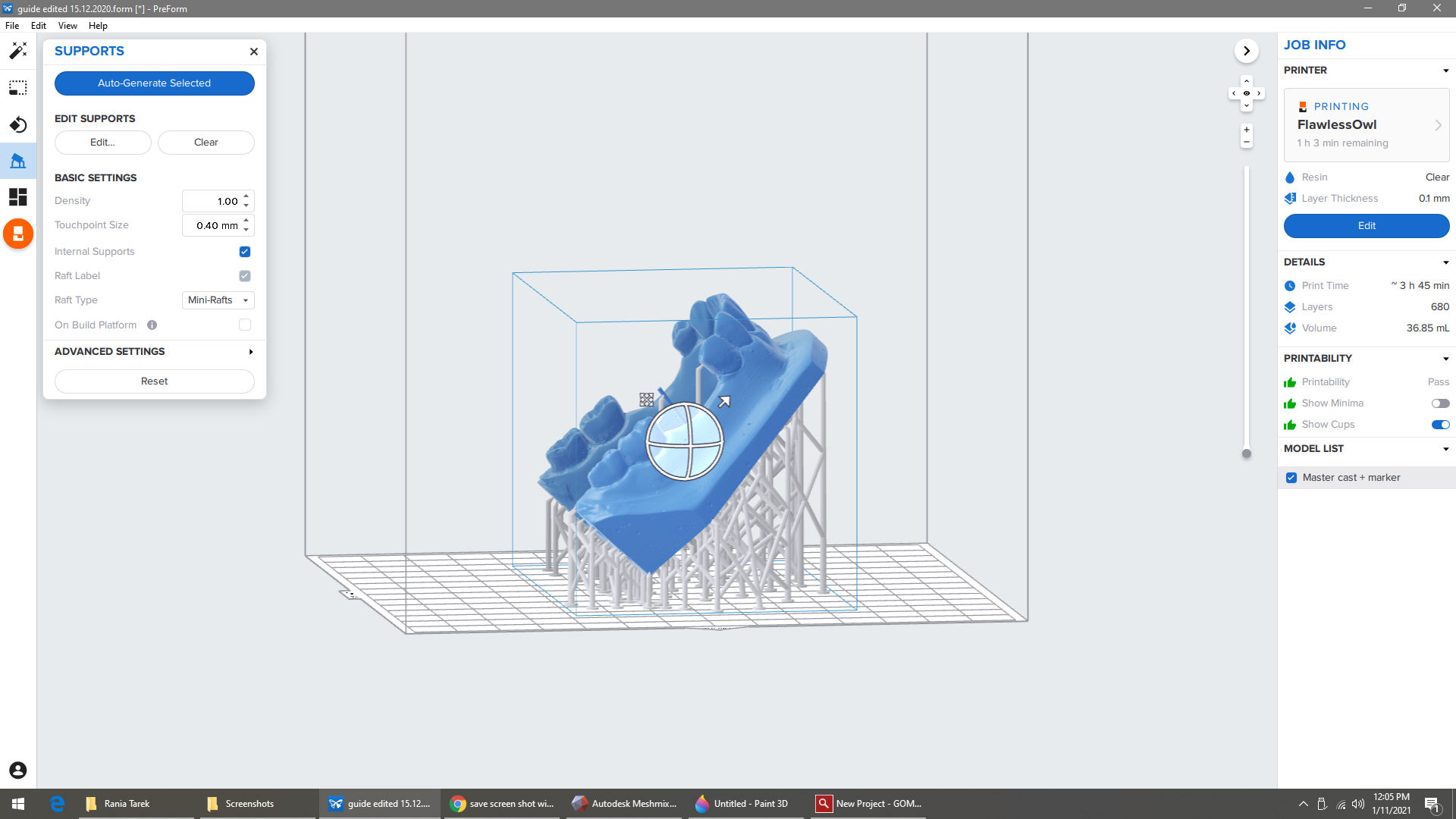

Supplement: Supplementary file 1 — Additional file 1. Study data. [file 12903_2022_2671_MOESM1_ESM.zip › additional file/Printing Process/Screenshot (3).png]

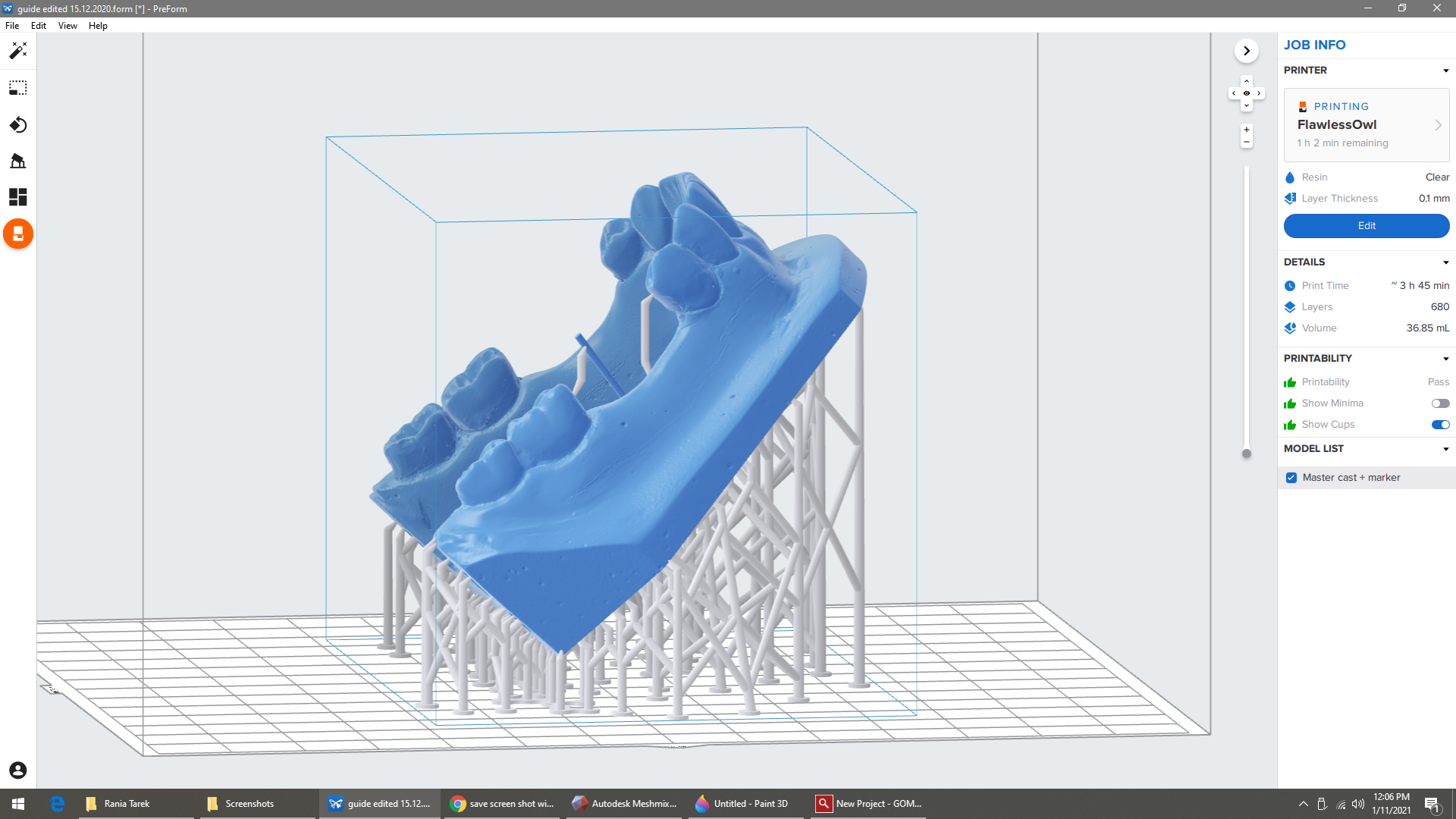

Supplement: Supplementary file 1 — Additional file 1. Study data. [file 12903_2022_2671_MOESM1_ESM.zip › additional file/Printing Process/Screenshot (4).png]

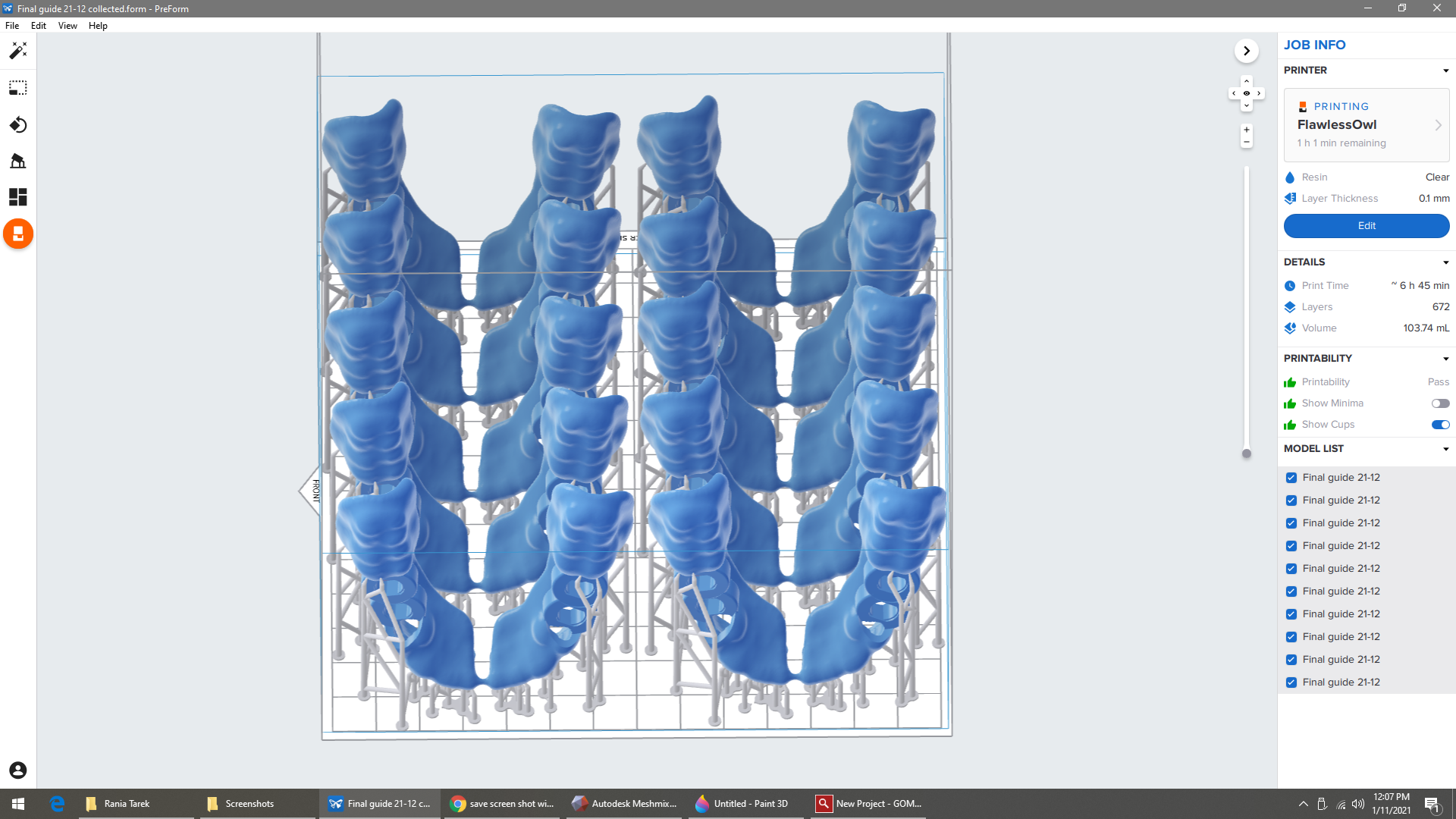

Supplement: Supplementary file 1 — Additional file 1. Study data. [file 12903_2022_2671_MOESM1_ESM.zip › additional file/Printing Process/Screenshot (5).png]

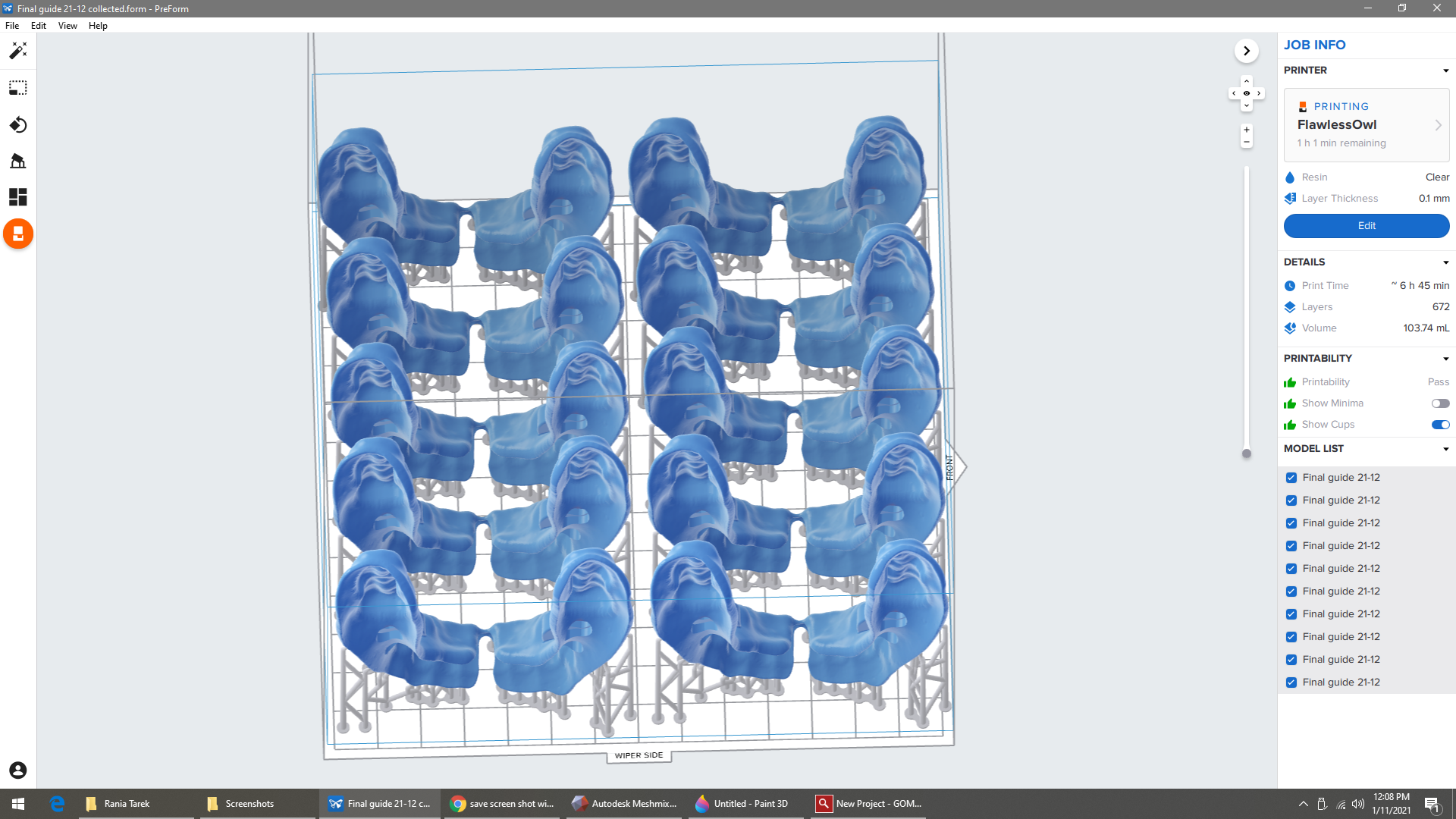

Supplement: Supplementary file 1 — Additional file 1. Study data. [file 12903_2022_2671_MOESM1_ESM.zip › additional file/Printing Process/Screenshot (6).png]

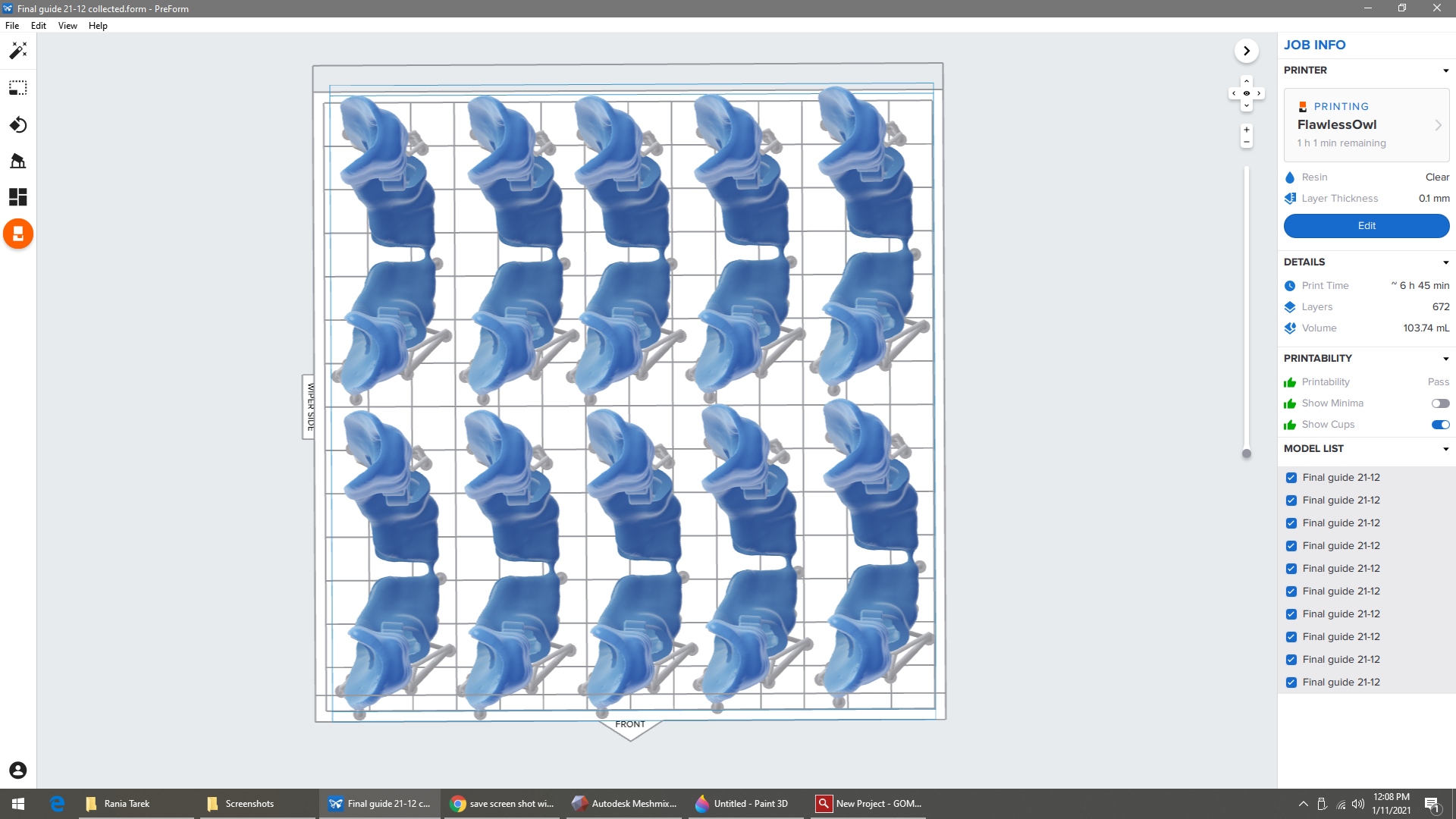

Supplement: Supplementary file 1 — Additional file 1. Study data. [file 12903_2022_2671_MOESM1_ESM.zip › additional file/Printing Process/Screenshot (7).png]

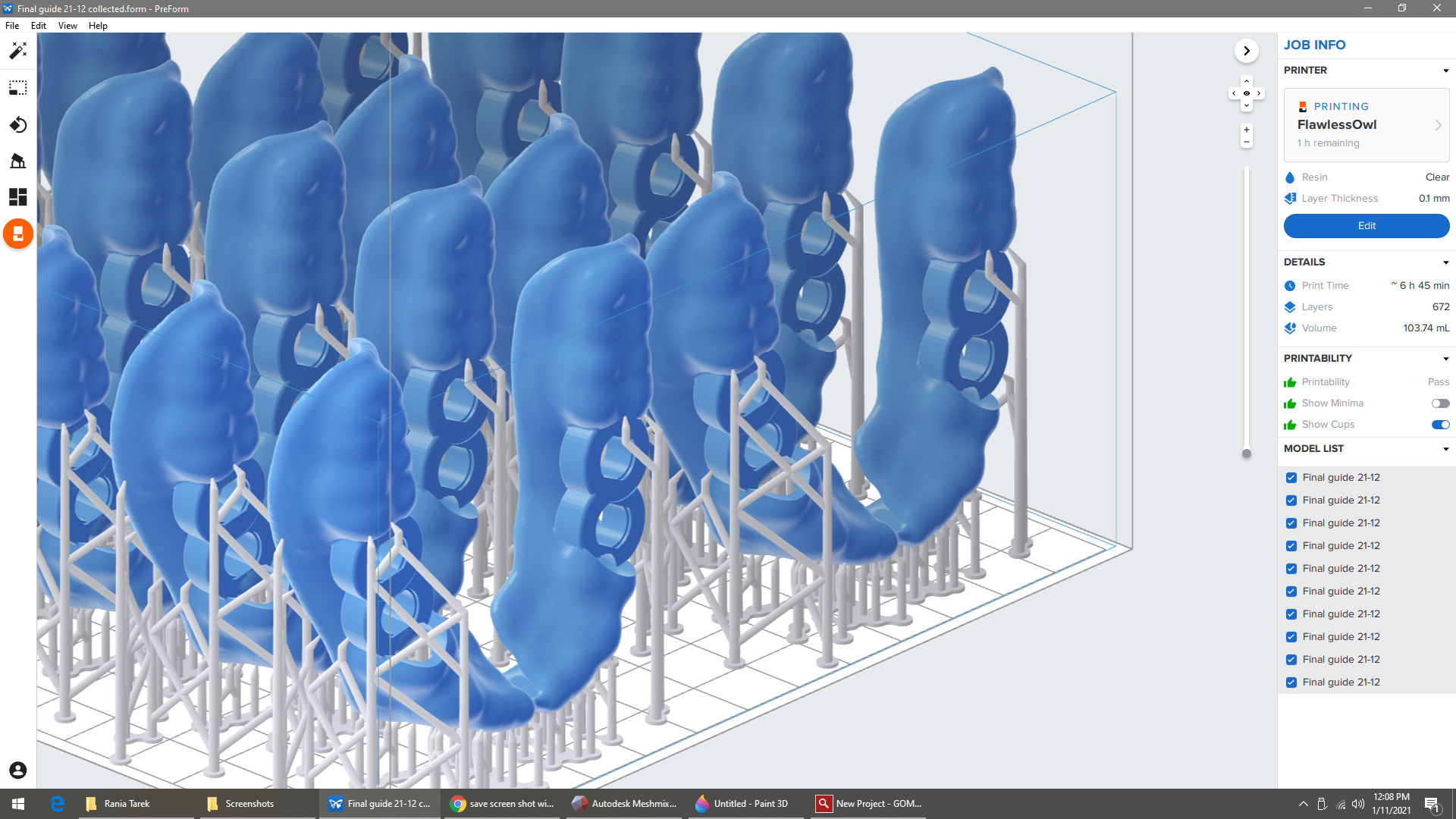

Supplement: Supplementary file 1 — Additional file 1. Study data. [file 12903_2022_2671_MOESM1_ESM.zip › additional file/Printing Process/Screenshot (8).png]

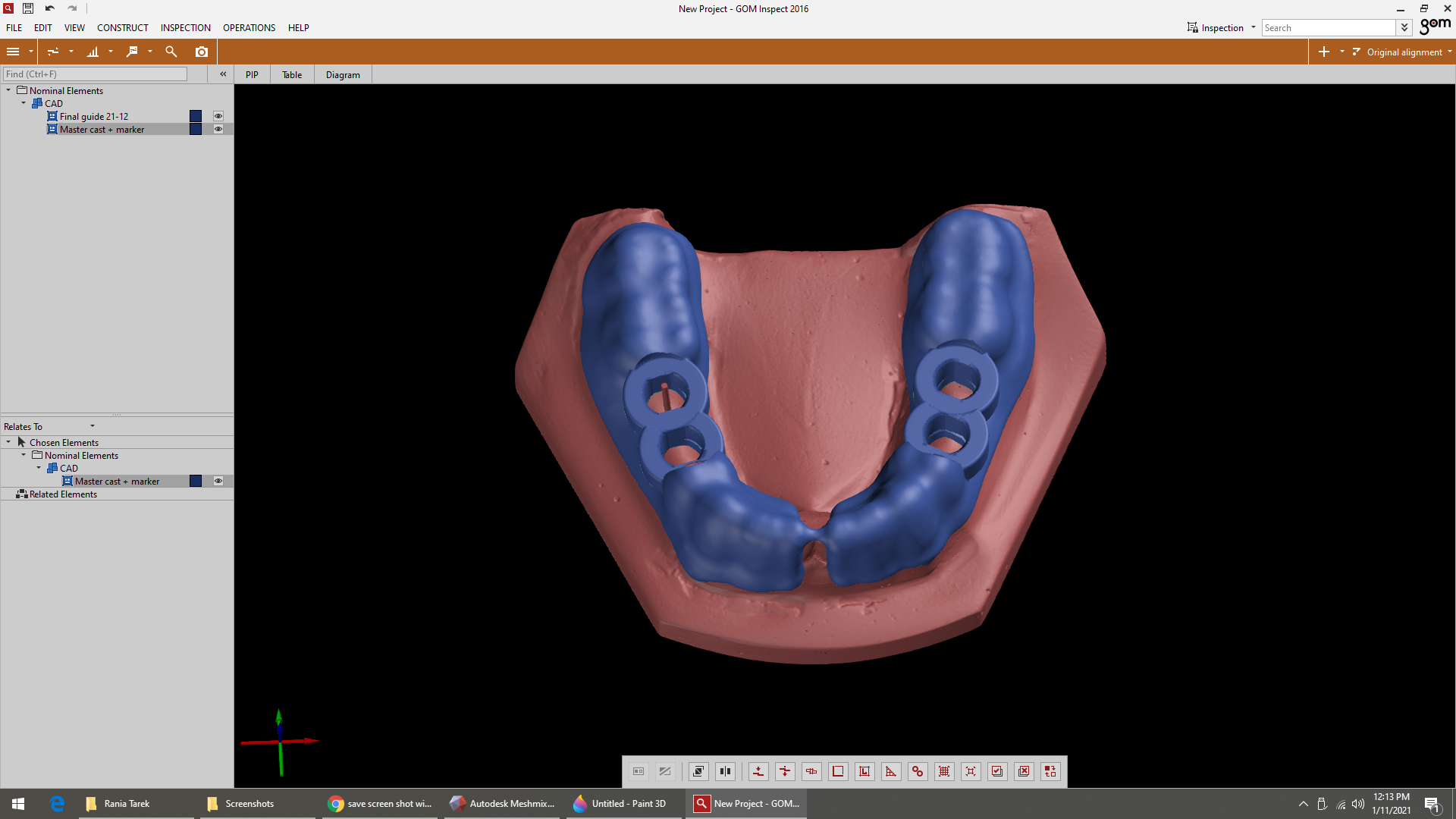

Supplement: Supplementary file 1 — Additional file 1. Study data. [file 12903_2022_2671_MOESM1_ESM.zip › additional file/Printing Process/Screenshot (9).png]
